# Phylogenetic comparisons reveal mosaic histories of larval and adult shell matrix protein deployment in pteriomorph bivalves

**DOI:** 10.1038/s41598-020-79330-x

**Published:** 2020-12-17

**Authors:** Ran Zhao, Takeshi Takeuchi, Ryo Koyanagi, Alejandro Villar-Briones, Lixy Yamada, Hitoshi Sawada, Akito Ishikawa, Shunsuke Iwanaga, Kiyohito Nagai, Yuqi Che, Noriyuki Satoh, Kazuyoshi Endo

**Affiliations:** 1 Department of Earth and Planetary Science, Graduate School of Science, University of Tokyo, Bunkyo-ku, Tokyo, 113-0033 Japan; 2 Marine Genomics Unit, Okinawa Institute of Science and Technology Graduate University, Onna, Okinawa, 904-0495 Japan; 3 DNA Sequencing Section, Okinawa Institute of Science and Technology Graduate University, Onna, Okinawa, 904-0495 Japan; 4 Instrumental Analysis Section, Okinawa Institute of Science and Technology Graduate University, Onna, Okinawa, 904-0495 Japan; 5 Sugashima Marine Biological Laboratory, Graduate School of Science, Nagoya University, Sugashima, Toba 517-0004 Japan; 6 Nagasaki Prefectural Institute of Fisheries, Nagasaki, Nagasaki 851-2213 Japan; 7 Pearl Research Institute, Mikimoto Co., Ltd, Shima, Mie 517-0403 Japan; 8 Department of Biology, Shenzhen MSU-BIT University, 1 International University Park Road, Dayun New Town, Longgang District, Shenzhen, Guangdong Province People’s Republic of China

**Keywords:** Evolutionary theory, Molecular evolution, Proteomics, Mineralogy

## Abstract

Molluscan shells are organo-mineral composites, in which the dominant calcium carbonate is intimately associated with an organic matrix comprised mainly of proteins and polysaccharides. However, whether the various shell matrix proteins (SMPs) date to the origin of hard skeletons in the Cambrian, or whether they represent later deployment through adaptive evolution, is still debated. In order to address this issue and to better understand the origins and evolution of biomineralization, phylogenetic analyses have been performed on the three SMP families, Von Willebrand factor type A (VWA) and chitin-binding domain-containing protein (VWA-CB dcp), chitobiase, and carbonic anhydrase (CA), which exist in both larval and adult shell proteomes in the bivalves, *Crassostrea gigas* and *Pinctada fucata*. In VWA-CB dcp and chitobiase, paralogs for larval and adult SMPs evolved before the divergence of these species. CA-SMPs have been taken as evidence for ancient origins of SMPs by their presumed indispensable function in biomineralization and ubiquitous distribution in molluscs. However, our results indicate gene duplications that gave rise to separate deployments as larval and adult CA-SMPs occurred independently in each lineage after their divergence, which is considerably more recent than hitherto assumed, supporting the “recent heritage and fast evolution” scenario for SMP evolution.

## Introduction

The appearance of mineralized tissues, including calcium phosphate, calcium carbonate, and silica, opened the grand history of metazoan taxa at the dawn of the Cambrian^1–3^. Among metazoan biominerals, calcium carbonate shells of the Mollusca provide exceptional resources for studying the processes of biomineralization due to their tremendously varied morphologies^4^, as well as a huge diversity of special microstructures, characteristic of each species^5,6^. Despite this complexity, adult molluscan shells are secreted by an evolutionarily homologous organ known as the mantle^7,8^, and different aspects of the shell formation processes are controlled by the organic molecules collectively known as the shell matrix. As additional shell matrix proteins (SMPs) are identified in different molluscs, lineage-specific repertoires of SMPs^9,10^, which control formation of prism and nacre^11,12^ and which govern larval and adult shell formation^13^, have been recognized. Meanwhile, only a few SMPs are apparently shared among different species. The rare SMPs that are shared by gastropods and bivalves include carbonic anhydrase (CA), blue mussel shell protein (BMSP), Perlucin, and Perlwapin^9,10,13^.

In order to explain evolutionary relationships among SMP repertoires and how their components were recruited for use in shell construction, two extreme scenarios have been proposed^14^. One, the “ancient heritage” scenario, is generally favored by the fossil record, with skeletal elements suddenly appearing in Tommotian rocks. The fossil record suggests that representatives of dominant mollusc classes appeared in the Cambrian, including polyplacophores, monoplacophores, cephalopods, gastropods, and bivalves^15,16^, some of which possessed complex shell microstructures^17^. A single, ancient origin of common SMPs is suggested by the rapid exploration of textural combinations, and most of the design possibilities for building exoskeletons^18^ resulted from recruitment of Precambrian gene functions that were not related to mineralization^19^. In addition, the primary structures of SMPs may also support speculation regarding the antiquity of some SMPs. For instance, great similarities between the functional domains of molluscan CAs, including SMPs for Nacrein^20^ and N66^21^, and CAs of other metazoans have been reported. Because the conversion of carbon dioxide into bicarbonate is simple inorganic chemistry, this function could be primordial in calcium carbonate biomineralization. Carbonic anhydrase domains have been found in both bivalves and gastropods, so it is impossible that such a key function resulted from recent recruitment^14^.

On the other hand, the “recent heritage and fast evolution” scenario is supported by unique origins of different shell matrices indicated by “independent inventions,” based on phylogenetic comparisons of homologous genes^14^. Transcriptomic data indicate that 85% of secreted proteins of the abalone, *Haliotis asinina*, are unknown, and only 19% of the secreted proteins of *H*. *asinina* are homologous to those of the patellogastropod, *Lottia scutum*^22^, suggesting that molluscan shells are constructed from rapidly evolving secretomes^14^. This scenario is also supported by the phylogenetic analysis of dermatopontins of eight gastropod species^23^. Dermatopontin is an ancient protein, found in various metazoans from sponges to humans^24–26^ with general roles in cell–matrix interactions and matrix assembly^27,28^. However, in the two gastropod lineages, Basommatophora (pond snails) and Stylommatophora (land snails), recruitment of dermatopontin to the shell occurred twice, independently^23^.

Although adult molluscan shells show complex micro-textures and different mineralogies, molluscan larval shells have similar microstructures, and are almost entirely composed of aragonite^29–34^, implying that larval shells are evolutionarily highly conserved^35^. If common mineralogy and microstructures are assumed to be hallmarks of “primitive” shells, studies of larval SMPs could help reconstruct ancestral features of larval shells, as well as the origin of SMPs in different lineages.

With the help of high-throughput DNA sequencing and proteomic techniques, we reported the first larval shell proteomes of two pteriomorph bivalves, the Pacific oyster, *Crassostrea gigas*, and the pearl oyster, *Pinctada fucata*^13^. Three protein families, including Von Willebrand factor type A (VWA) and chitin-binding domain-containing protein (VWA-CB dcp), CA and chitobiase, were identified in both larval and adult shells of the two species^13^. Since those three SMPs exist in both larval and adult shells in both species, they are likely to be functionally important^13^. Moreover, they allow us to examine when dual roles as larval and adult SMPs evolved, relative to the divergence of those species. In this study, phylogenetic analyses have been performed on these SMP families with the aim of inferring when they were recruited as SMPs to larval and adult shells.

## Results

### Evolutionary history of molluscan VWA-CB dcps

**Figure 1:**
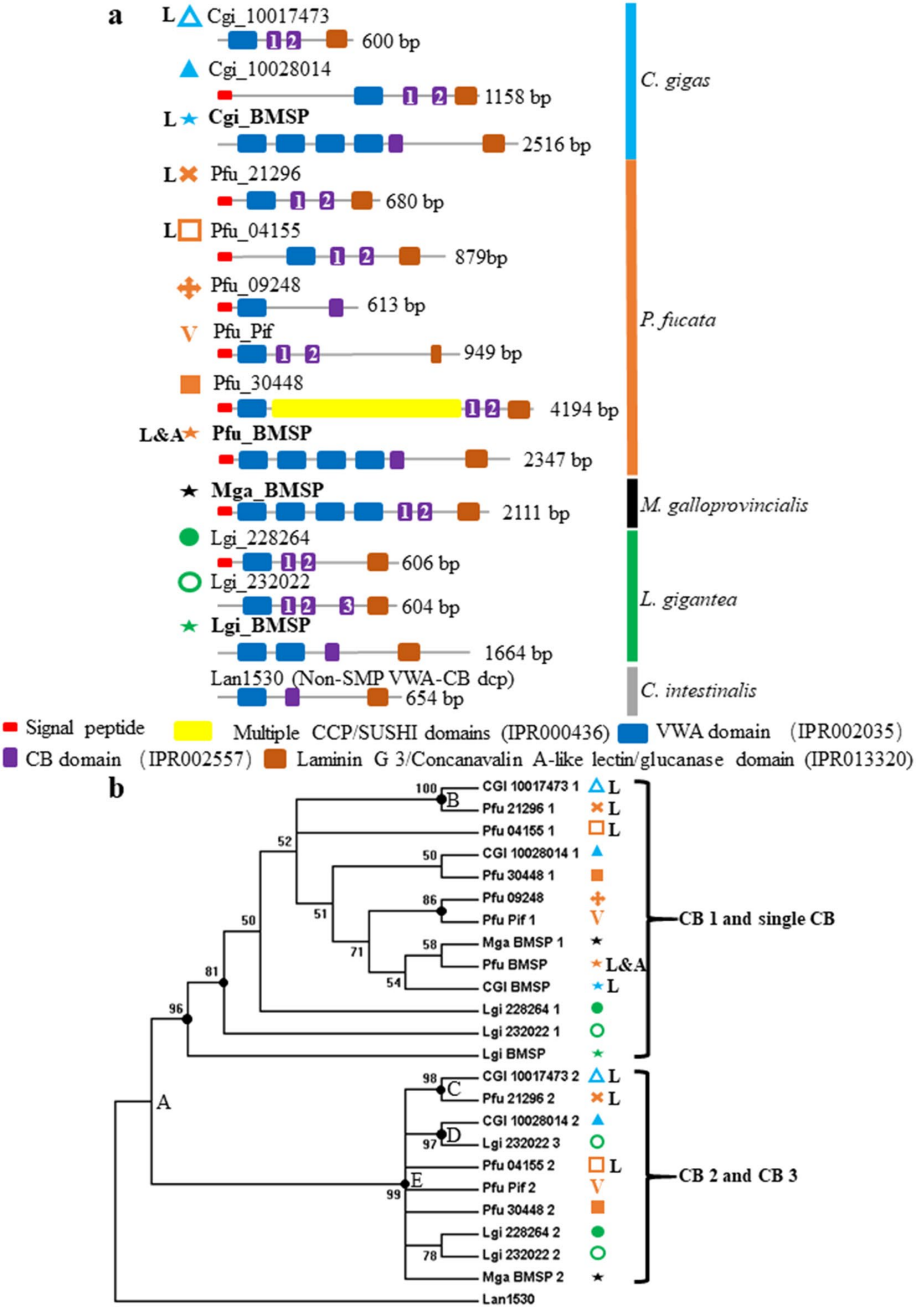
Phylogenetic analyses of CB domains of VWA-CB dcps of molluscan shells. (**a**) Schematic representations of the domain structures of shell-specific VWA-CB dcps. (**b**) A Bayesian tree based on LG model and 84 amino acid residues. Polychotomy is generated if the posterior probability value of the node is < 50. Posterior probability values are shown if ≥ 50, and marked with black dots if ≥ 80. The same symbols denoting different proteins in panel a are used in panel b. Larval SMPs are marked by “L”, and Pfu-BMSP identified in both larval and adult shells of *P. fucata* is marked by “L&A”. Cgi, *Crassostrea gigas*; Pfu, *Pinctada fucata*; Mga, *Mytilus galloprovincialis*; Lgi, *Lottia gigantea*; Lan, *Lingula anatina*.

**Figure 2:**
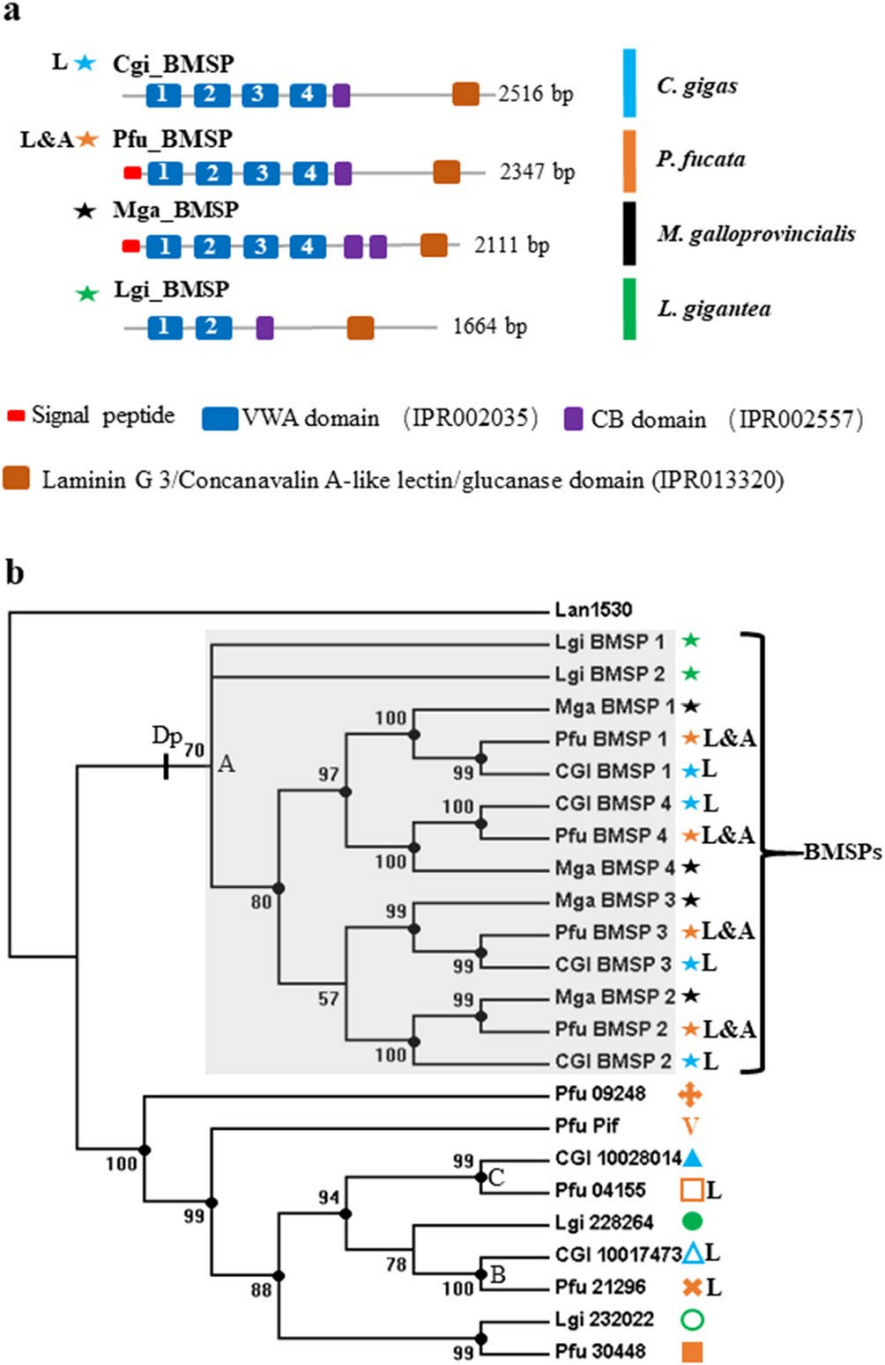
Phylogenetic analyses of VWA domains of VWA-CB dcps of molluscan shells. (**a**) Schematic representations of domain structures of shell-specific BMSPs. (**b**) A Bayesian tree based on the LG model and 187 amino acid residues. Polychotomy is generated if the posterior probability value of the node is < 50. Posterior probability values are shown if ≥ 50, and marked with black dots if ≥ 80. The same symbol marks as in Fig. 1 are used to denote different SMPs. Larval SMPs are marked by “L”, and Pfu-BMSP identified from both larval and adult shells of *P. fucata* is indicated by “L&A”. BMSP family proteins are indicated by bold characters. Cgi, *Crassostrea gigas*; Pfu, *Pinctada fucata*; Mga, *Mytilus galloprovincialis*; Lgi, *Lottia gigantea*; Lan, *Lingula anatina*; Dp, duplication.

According to a genome-wide survey based on the InterProScan online database (Supplementary table S1), as well as our proteomic work, proteins possessing both VWA and CB domains appeared in the common ancestor of Mollusca and closely-related lophotrochozoans including the Nemertea, Phoronida, and Brachiopoda. VWA-CB domain-containing protein family expanded exclusively in the molluscan lineage^13^. As molluscan SMPs, VWA-CB dcps have been reported from the shells of *Mytilus galloprovincialis*^36^, *Lottia gigantea*^9,37^ and in both the larval and adult shells of *Crassostrea gigas* and *Pinctada fucata*^13,38,39^. Three major groups of VWA-CB dcps can be distinguished: (1) SMPs with a single VWA domain and one or more (typically two) CB domains, such as Pif, which participates in nacre formation in *Pinctada fucata*^38^. (2) BMSP, which is an SMP with multiple (typically four) VWA domains and one or two CB domains. These have been identified from both bivalves and gastropods^9,13,36^, although some researchers regard BMSP proteins as a subgroup of Pif^40^. (3) Other non-SMP VWA-CB dcps, typically with a single VWA domain and one or more (typically two) CB domains. SMP VWA-CB dcps also typically contain a laminin G (LG) domain^40^. In this study, mollucan VWA-CB dcps identified from shell matrix extracts (Figs. 1a and 2a; supplementary table S2) were subjected to phylogenetic analyses. CB domains (Fig. 1a) and VWA domains (Fig. 2a) are numbered from the N-terminus to the C-terminus.

Also known as the cellulose-binding domain, the CB domain is found in carbohydrate-active enzymes, and its deployment is significantly expanded in Mollusca, Brachiopoda and Arthropoda^13,41,42^ (Supplementary table S1). In the phylogenetic trees of CB domains (Figs. 1b; S1a, b, c, d and e), the first CB domain of the VWA-CB dcps with multiple CB domains and the CB domain of the VWA-CB dcps with single CB domain form a monophyletic group. The first CB domains of BMSPs are also nested within the group (Figs. 1b; S1a, b, c, d and e). The second and the third CB domains of the VWA-CB dcps with multiple CB domains comprise another monophyletic group (Figs. 1b; S1b, c and e). The larval VWA-dcps of *C. gigas* and *P. fucata* cluster in a group at nodes B and C (Figs. 1b; S1a, b, c, d and e). Amongst trees built via different algorithms, Baysian trees exhibited almost identical topology as ML trees. While between MEGA and PhyML, the trees generally exhibited very similar topologies, and the bootstrap values were also comparable.

The occurrence of multiple VWA domains distinguishes BMSPs from other VWA-CB dcps by forming a unique cluster (Figs. 2b; S2a, b, c and d; S3b, c, d and e). Therefore, duplication (Dp) events of the VWA domain generated multiple VWA domains of BMSPs in the common ancestor of bivalves and gastropods before their divergence can be inferred (Figs. 2b; S2a, b and c; S3b, c, d and e, node A). Recruitment of VWA-CB dcps to the larval shell of the common ancestor of *C. gigas* and *P. fucata* before speciation is also suggested by node B (Figs. 2b; S2a, b, c and d; S3a, b, c, d and e), where larval SMPs of both species form a single group.

**Figure 3:**
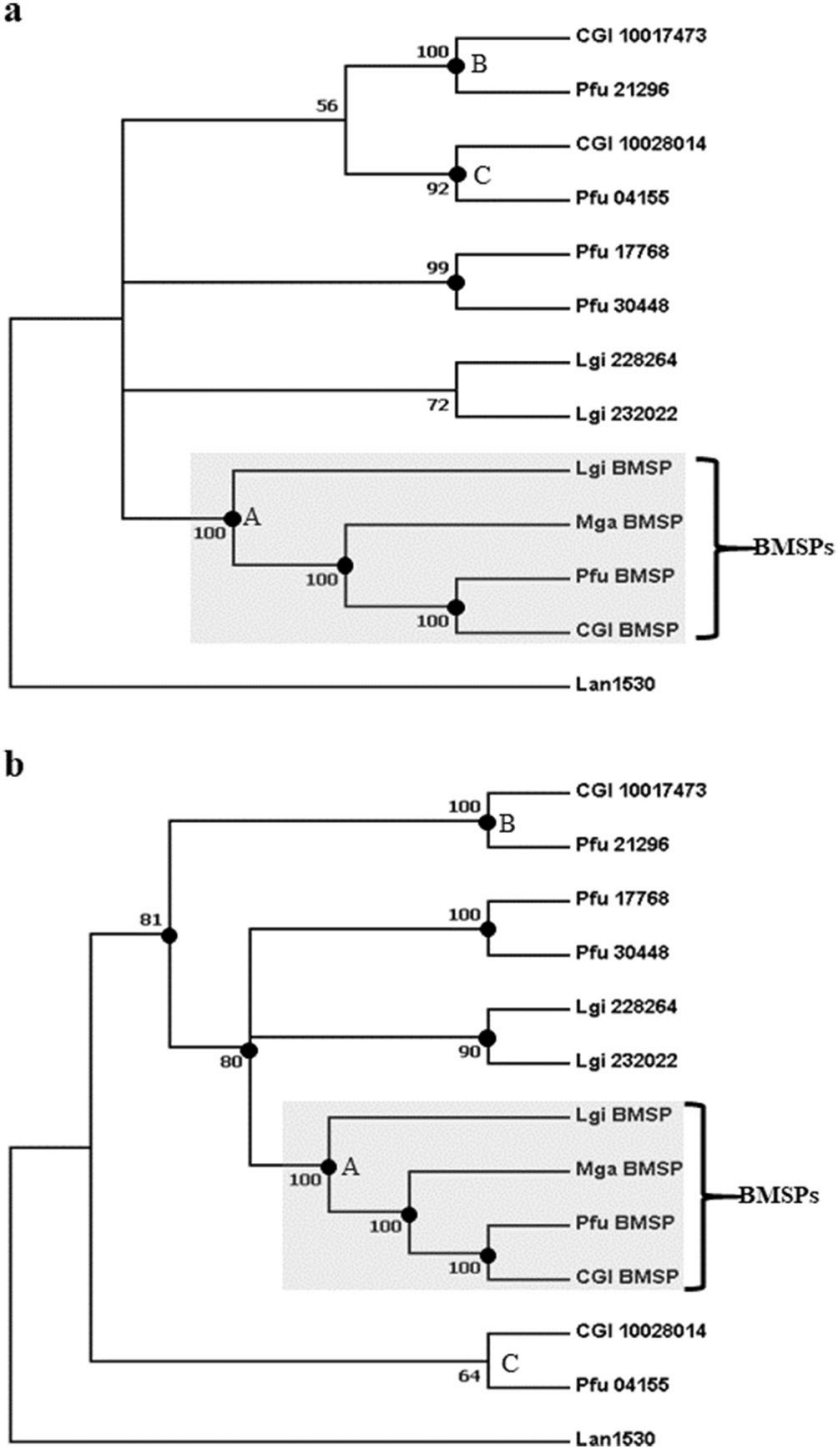
Phylogenetic analyses of Laminin G domains on 210 amino acids and the concatenated sequence of a CB domain and the Laminin G domain on 282 amino acids of VWA-CB dcps of molluscan shells. (**a**) A Bayesian phylogenetic tree based on Laminin G domains. (**b**) A Bayesian phylogenetic tree based on concatenated sequences of the CB and Laminin G domains. Polychotomy is generated if the posterior probability of the node is < 50. Posterior probabilities are shown if ≥ 50, and marked with black dots if ≥ 80. Cgi, *Crassostrea gigas*; Pfu, *Pinctada fucata*; Mga, *Mytilus galloprovincialis*; Lgi, *Lottia gigantea*; Lan, *Lingula anatina*. The group formed by BMSPs is indicated.

Laminin G domains have been reported from the region downstream of the chitin-binding domain in Pif/BMSP-like proteins^40^. In this study, a combined domain search using SMART and BLAST revealed a conserved Laminin G-like region from the VWA-CB dcps, with the exception of Pfu_09248 (Fig. 1a). Phylogenetic analyses were performed on the Laminin G domain sequences (Figs. 3a; S4a, b, c and d). Because the CB1 and the CB domain of the VWA-CB dcps with single CB were inferred to be orthologues (Figs. 1b; S1a, b, c, d and e), the concatenated sequences of the CB domain and Laminin G domain regions were also subjected to phylogenetic analyses (Figs. 3b; S5a, b, c and d). Again, the topology suggested recruitment of the VWA-CB dcp to the larval shell of the common ancestor of *C. gigas* and *P. fucata* before their divergence (Figs. 3a and b, node B; S4a, b, c and d, node B; S5a, b, c and d, node B), and duplication of the VWA domain in the common ancestor of bivalves and gastropods gave rise to BMSPs in the shell, as revealed by monophyly of BMSPs (Figs. 3a and b; S4a, b, c and d; S5a, b, c and d).

### Chitobiases were present in larval and adult shells of the last common ancestor of *C. gigas* and *P. fucata*

**Figure 4:**
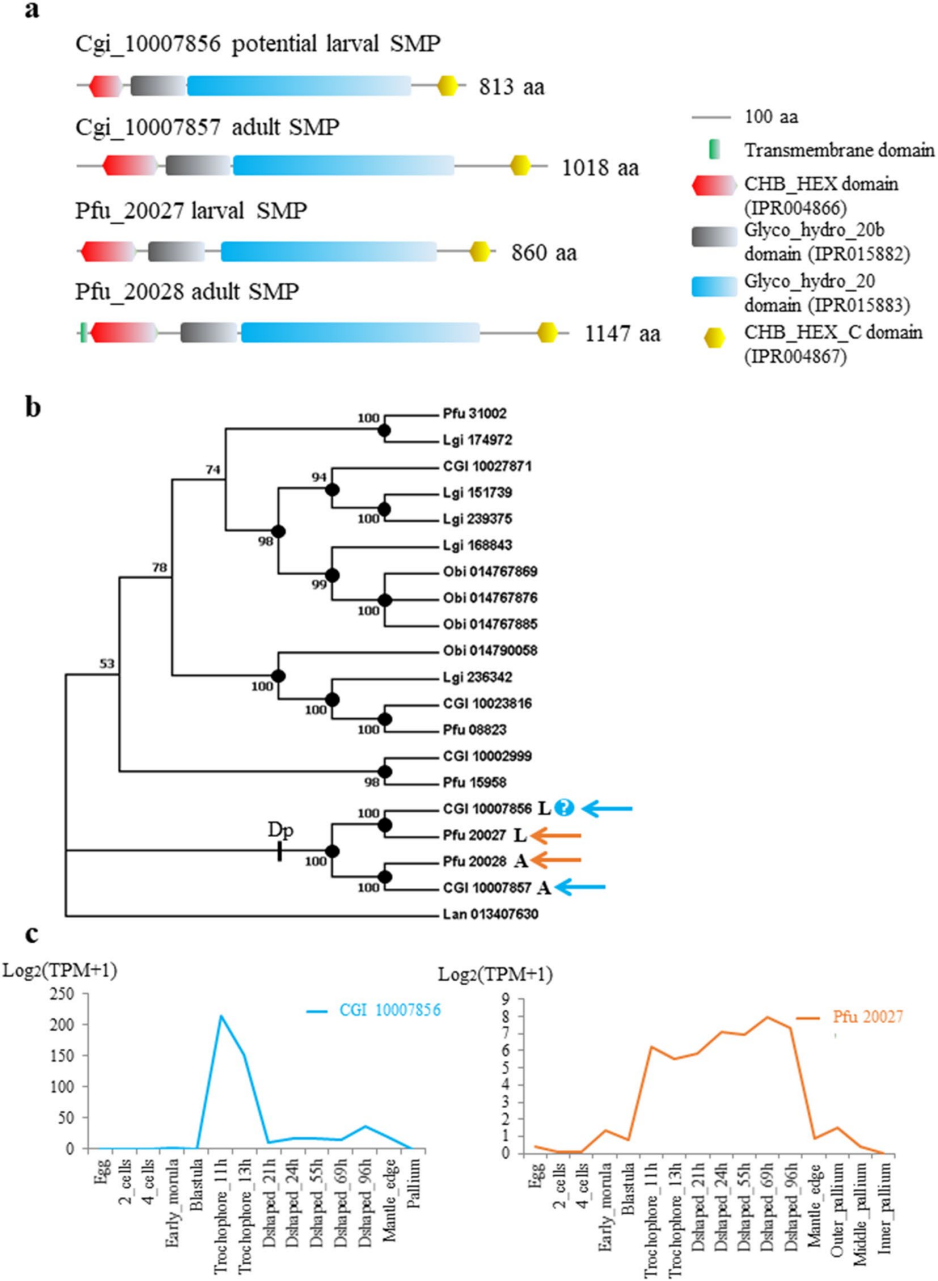
Phylogenetic analyses of chitobiases in molluscs on 1066 amino acid residues. (**a**) Schematic representations of the domain structures of chitobiases. (**b**) Bayesian phylogenetic estimation for chitobiases in molluscs using the concatenated sequences of CHB_HEX domain (IPR004866), Glyco_hydro_20b domain (IPR015882), Glyco_hydro_20 domain (IPR015883) and CHB_HEX_C domain (IPR004867). (**c**) Stage-specific expression of the larval chitobiase during development in *C. gigas* (blue) and *P. fucata* (orange)^13,39,59^. Polychotomy is generated if the posterior probability value of the node is < 50. Posterior probability values are shown if ≥ 50, and marked with black dots if ≥ 80. SMPs are indicated by blue (*C. gigas*) and orange (*P. fucata*) arrowheads. The question mark indicates that whether or not the gene is encoding an SMP is uncertain. Cgi, *Crassostrea gigas*; Pfu, *Pinctada fucata*; Lgi, *Lottia gigantea*; Obi*, Octopus bimaculoides.* Lan, *Lingular anatina*; Dp, duplication.

Chitin and fibroin-like proteins are considered integral to the shell matrix to provide the framework for nucleation and growth of crystals^43,44^. Chitinases and chitobiases have been reported from organic matrices of adult molluscan shells^13,37,39,45,46^. A chitobiase, Pfu_20027, was identified as a chitinolytic enzyme in the larval shell (Fig. 4a, supplementary tables S3 and S4)^13^ A BLASTP search of the *P. fucata* larval chitobiase against gene models of the whole genome of *C. gigas* revealed that the protein predicted from CGI_10007856 is highly similar (Identity: 70%; E-value: 0) to the larval shell-chitobiase of *P. fucata*^13^ (Fig. 4a), which suggests that this gene is a potential SMP that escaped detection by proteomic analyses. Molecular phylogenetic analyses indicated that the hypothetical larval SMP, CGI_10007856, indeed clustered within the group comprised of the other three shell-chitobiases, suggesting that it is likely an SMP of oyster larvae (Figs. 4b; S6a, b, c and d).

### Relatively recent recruitment of CAs to bivalve shells

**Figure 5:**
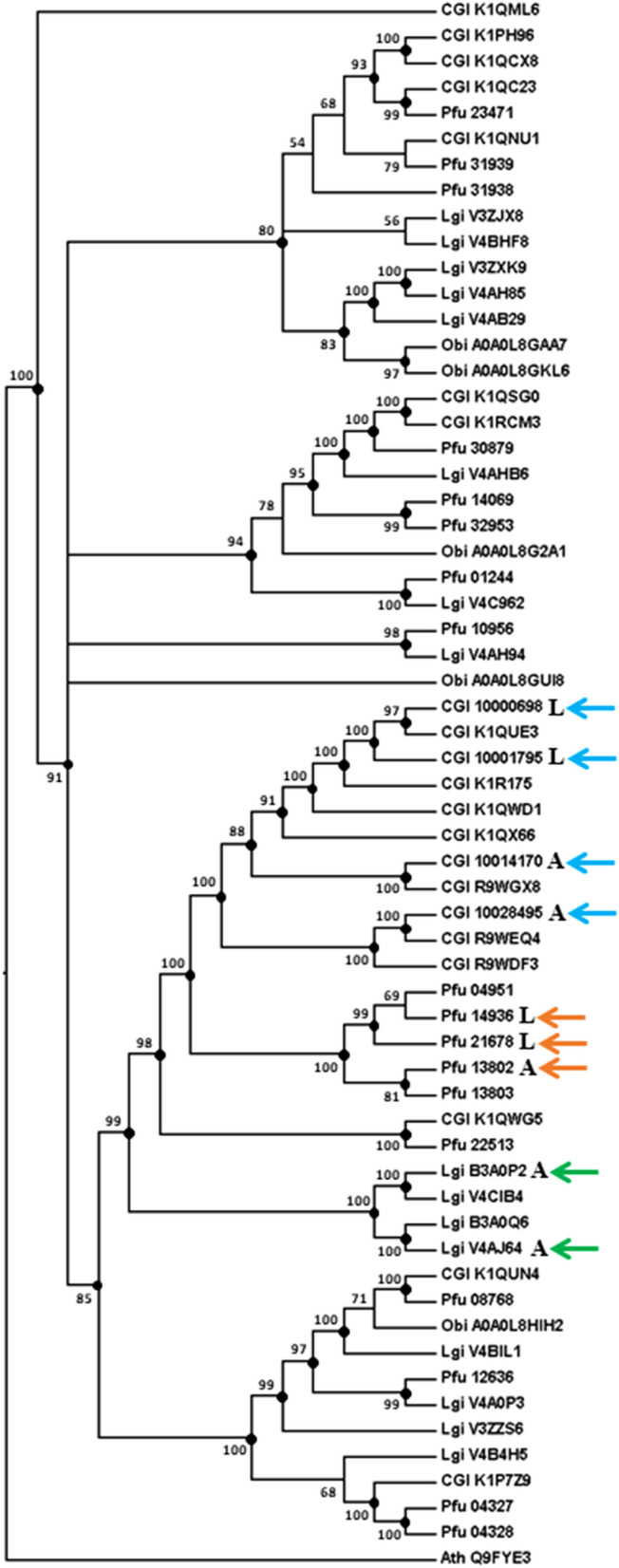
Bayesian phylogenetic analysis based on 935 amino acid residues of CA domains in molluscs. SMPs are indicated by blue (*C. gigas*), orange (*P. fucata*) and green (*L. gigantea*) arrowheads. Polychotomy is generated if the posterior probability of the node is < 50. Posterior probabilities are shown if ≥ 50, and marked with black dots if ≥ 80. Cgi, *Crassostrea gigas*; Pfu, *Pinctada fucata*; Lgi, *Lottia gigantea*; Obi*, Octopus bimaculoides.* Ath, *Arabidopsis thaliana*.

Conversion of carbon dioxide into bicarbonate is thought to be an ancestral function in carbonate biomineralization^14^. CAs have been reported from adult shells of various bivalves and gastropods^9,10,13,37,39^, as well as larval shells of bivalves^13^. The number of CA domains is highly expanded in molluscs (bivalves, gastropods, and cephalopods), compared to most other metazoan phyla^13^. Taken together, it is tempting to conclude that CAs in shells of extant molluscs were inherited from the molluscan common ancestor in the early Cambrian, before divergence of the main classes^15,47^. Surprisingly, however, CAs extracted from shells of *C. gigas*, *P. fucata*, and *L. gigantea* formed three separated clusters, each of which comprises proteins of one species only (Figs. 5; S7a, b, c and d; S8a, b, c, d and e). This indicated that recruitment of CAs as SMPs occurred independently in each molluscan species. In bivalves, recruitment is inferred to have occurred even after the divergence of *C. gigas* and *P. fucata* (Figs. 5; S7a, b, c and d; S8a, b, c, d and e). The CA SMPs and the CA of humans form separated clusters (Fig. S8a, b, c, d and e). Therefore, it is not certain that molluscan shell CAs exhibit more similarities to any human CAs than to any others. Meanwhile, phylogenetic analyses performed on molluscan CAs indicate that either larval or adult shell CAs could have been derived from duplication of the other.

## Discussion

The phylogenetic analyses performed on VWA-CB dcps indicate that all extant molluscan shell-specific VWA-CB dcps, including BMSPs, originated from an ancestor with two CB domains (Figs. 1b; S1b, c and e), while some of them lost the second and third CB domains during evolution. Recruitment of the VWA-CB dcp to the larval shell of the common ancestor of *C. gigas* and *P. fucata* before their divergence is indicated by the larval group of the two species at nodes B and C (Figs. 1b; S1a, b, c, d and e).

Phylogenetic analyses of CB, VWA, and Laminin G domains performed on VWA-CB dcps identified in shells of bivalves and gastropods all suggested that VWA-CB dcps, including BMSPs, were recruited by the common ancestor of bivalves and gastropods before their divergence, an assumption that is congruent with their conserved domain architecture and exclusive distribution in the Mollusca and related animal phyla, as indicated by our previous genome-wide survey^13^.

The VWA domain is thought to be involved in protein–protein interactions and is often found in extracellular proteins, such as plasma proteins, integrin, and collagen^48–50^. In the present study, phylogenetic analyses performed on VWA domains indicated BMSP genes are demonstrated to be orthologous, in contrast to the scenario indicated by a previous study^40^, in which multiple VWA domains of BMSPs were suggested to have been produced by independent duplications in each species. The previous study was based on an alignment between the BMSP of *M. galloprovincialis,* an SMP identified using a calcium carbonate-binding assay^36,40^, and Lgi_236719, a multiple VWA domain-containing protein deduced from the genome data of *Lottia gigantea* (v1.0, http://genome.jgi-psf.org/Lotgi1/Lotgi1.home.html). Indeed, phylogenetic analyses of VWA domains show this theoretical BMSP Lgi_236719 remains out of the cluster formed by BMSPs of other species (Figs. S3a, b, c, d and e), and form a single cluster with one or two *L. gigantea* VWA-CB dcps that exhibit a single VWA domain (Figs. S3a, c and e). This observation indicates that species-specific duplications of the VWA domains in Lgi_236719 are distinct from those of other BMSPs. However, it is dubious whether Lgi_236719 of *L. gigantea* is a shell protein, since its presence in the shell has not been confirmed by shell proteome analysis, and it is not found in the published shell proteome data of *L. gigantea*^9^. Therefore, it appears possible that the hypothesis proposed in a previous study^40^ was built on a comparison between SMPs and a non-SMP with other physiological functions.

The BMSP of *M*. *galloprovincialis* possesses two CB domains, and the second CB domain clustered with the monophyletic group of CB 2 domains of other VWA-CB dcps. This indicates that BMSP originally also had two CB domains, like other VWA-CB dcps in the ancestral protein at node A (Figs. 1b; S1a, b, c, d and e). It is inferred that the CB 2 domain has been lost in BMSPs of *C. gigas*, *P. fucata* and *L. gigantea*, rather than acquired only in *M*. *galloprovincialis.*

In *L. gigantea*, CB 2 of Lgi_232022 is orthologous to CB 2 of Lgi_228264, forming a clade (Figs. 1b; S1a, b, c and e), and CB3 of Lgi_232022 is located in the group with CB2 domains (Figs. 1b; S1b, c and e), indicating a duplication event of the protein within gastropods. CB3 is probably duplicated from the CB2 in Lgi_232022. Thus, the cluster comprised of CB 3 of Lgi_232022 and CB 2 of CGI_10028014 at node D (Figs. 1b; S1a, b, c, d and e) may be a result of a long branch attraction, because node E (Figs. 1b; S1b, c and e) is not supported by very high bootstrap values and those branches appear to be longer than others (Fig. S1c).

Notably, unlike the other larval SMP, Pfu_21296, which forms a clade with the larval SMP, CGI_10017473 (Figs. 1b and S1a, b, c, d and e, nodes B and C; Figs. 2b and S2a, b and d, node B), as a larval SMP, Pfu_04155 forms a clade with an adult SMP of *C. gigas*, CGI_10028014 (Figs. 2b, S2b and d; S3a, node C). Thus, this larval SMP, Pfu_04155, is suggested to be derived from an ancestral adult SMP, or that the ancestral larval common ancestor gave rise to an adult SMP. Interestingly, in contrast to other VWA-CB domain-containing SMPs of *P. fucata*, it exhibits an expression pattern with double peaks, being highly expressed in both larval and adult stages in the pearl oyster (Fig. S2e), suggesting that it retains a transitional phase in its history when an original adult shell protein was recruited by the larval SMP repertoire later for formation of the larval shell, or vice versa. The genetic mechanism behind this hypothesis might be explained in terms of heterochronic gene expression, implying that characterizations of developmental gene networks controlling formation of larval and adult shells may help to solve this question of the antiquity of larval shells.

The group comprising larval chitobiases of *C. gigas* and *P. fucata* and the group of adult chitobiases of *C. gigas* and *P. fucata*, supported by topologies of all trees, explicitly suggests that a duplication (Dp) event occurred in the chitobiase in the larval or adult shell of the last common ancestor of both species before their divergence (Figs. 4b; S6a, b, c and d). Thus, recruitment of chitobiases to larval and adult shells of the last common ancestor of *C. gigas* and *P. fucata* seems reasonable.

Transcriptomic data showed that expression of CGI_10007856 peaks at the trochophore stage, when the larval shell starts to form. Compared with that of the larval shell-chitobiase of *P. fucata*, a sudden reduction in expression with the formation of D-shape larva was observed (Fig. 4c), which may explain why it was undetectable in the larval shell proteome.

This study discovered independent recruitments of CAs to the shells of *C. gigas* and *P. fucata* after their divergence, an event that is estimated to have occurred in the Triassic or the Silurian at the earliest, depending on the phylogenetic interpretation of fossil taxa^51^, or during the period from the Carboniferous to the Triassic, based on the molecular clock^46^. In either scenario, functional diversification of CA-SMPs in larval and adult shells of those two bivalves was more recent than expected. Nonetheless, the topology of phylogenetic analyses agreed with two previous opinions^13^: 1. lineage-specific gene family expansion of CAs of bivalves and gastropods is supported by clusters consisting entirely of CAs of bivalves or of gastropods; 2. multiple homologs of CAs in larval or adult molluscan shells were produced by independent duplications of CAs within each species (Figs. 5; S7a, b, c and d).

As the hydrolytic enzyme of carbon dioxide in the Eq. (1),

1 $$ {\text{CO}}_{2} \, + \,{\text{H}}_{2} {\text{O}}\, \to \,{\text{HCO}}_{3}^{ - } \, + \,{\text{H}}^{ + }  $$

CA is important for providing HCO_3_^−^ that reacts with Ca^2+^ to form CaCO_3_. CA is well expanded in molluscan species as well as in other metazoan taxa that produce calcium carbonate skeletons^13^. Therefore, it is surprising to see that after divergence of the ancestors of *C. gigas* and *P. fucata*, a single CA gene, which may or may not have encoded an SMP, gave rise to multiple copies of CA genes in each lineage, some of which were deployed as adult SMPs, while others were deployed as larval SMPs in each lineage. Therefore, although CA was once taken as evidence to support the “ancient heritage” scenario of the origin of calcification of molluscs^14^, it actually supports the “recent heritage and fast evolution” scenario^14^, based on results from this study.

As a characteristic of nacrein and N66 proteins of *Pinctada* species^52^, the NG-repeat region was demonstrated in vitro to inhibit CaCO_3_ precipitation^53^. An important role of the NG-repeat domain for the inter-molecular interactions in the biomineralization processes^20,21,52–54^ and its calcium-binding ability has also been suggested, although the latter role is still under debate^20,54^. However, the NG-repeat domain was identified from neither the larval nacrein protein of *P. fucata* nor from the larval or adult nacreins of *C. gigas* (Fig. S9). The absence of this domain suggests possible functional divergence of nacrein proteins between larval and adult SMPs in *P. fucata* and between *C. gigas* and *P. fucata*.

In summary, deeply phylogenetic analyses on three SMP families, which may play essential roles in the formation of larval and adult shells of *Crassostrea gigas* and *Pinctada fucata*, provided insight into the ancient characters of SMPs in their ancestral larval and adult shells, and when duplications of these genes occurred relative to the specification of the two species in their evolutionary histories. The independent deployment of CA-SMPs indicated they might have indispensable functions in shell formation process in each species.

## Methods

### Data resources

Details of shell matrix proteins (SMPs) of *Crassostrea gigas* and *Pinctada fucata*, including amino acid sequences and results of annotation can be found in our previously published report^13^. Gene expression patterns of the two species included in this study (Figs. 4c and S2e) are based on the RNA-Seq data published by previous genomic studies. Briefly, total RNA of *P. fucata* was extracted from adult mantle tissues and 12 developmental stages from the egg to D-shaped larva. RNA-seq libraries were prepared using a TruSeq RNA sample Prep Kit v2 (Illumina) and sequenced with the Illumina GAIIx platform. For *C. gigas*, RNA-Seq data was retrieved from GigaDB (http://gigadb.org/) (Zhang et al. 2012). In order to analyze gene expression levels, TPM (transcripts per kilobase million) were calculated using eXpress 1.5.1^55^. Details of shell matrix proteins (SMPs) of *Lottia gigantea* are obtained from previous shell proteomic studies on the species^9,37^. Amino acid sequences and other details of other proteins of species (*Lottia gigantea*, *Mytilus galloprovincialis, Lingula anatina, Octopus bimaculoides*, *Homo sapiens* and *Arabidopsis thaliana*) were obtained from public databases, including InterPro protein analysis and classification (http://www.ebi.ac.uk/interpro/), Genebank (https://www.ncbi.nlm.nih.gov/genbank/), *Pinctada fucata* genome project (http://marinegenomics.oist.jp/pearl/viewer/info?project_id=36) (Supplementary tables S2, 3, 4 and 5).

### Sequence analysis

Blastp searches against the UniProtKB/Swiss-Prot database (https://blast.ncbi.nlm.nih.gov/Blast.cgi) were performed using the SuperComputer facilities of the National Institute of Genetics (NIG) with default settings. SMART online service (http://smart.embl-heidelberg.de) and BLAST were employed to predict the presence of functional domains and signal peptides.

### Alignments and settings for phylogenetic trees

In order to preserve the phylogenetically informative sites as much as possible, whole sequence of domains were subjected to multiple alignment using MUSCLE online service (https://www.ebi.ac.uk/Tools/msa/muscle/). Alignment of all datasets were deposited in TreeBASE (http://purl.org/phylo/treebase/phylows/study/TB2:S25319). Bayesian analysis was performed using the web service of MrBayes version 3.2.6^56^ supplied on Phylogeny.fr homepage (http://www.phylogeny.fr/index.cgi). We use the likelihood model with default settings (Number of substitution types: GTR; Substitution model: Poisson; Rates variation across sites: Invariable + gamma). Markov Chain Monte Carlo (MCMC) parameters were set to sample a tree every 10 or 100 generations of 10^5^ generations and the burn-in was set at 250 trees sampled. Resulted tree was accepted when the average standard deviation of split frequencies is lower than 0.05 and the average Potential Scale Reduction Factor (PSRF) for parameter values is around 1.0 (0.9–1.1), simultaneously. Furthermore, we performed maximum-likelihood estimation of phylogeny on MEGA X^57^. Evolutionary model and rates among sites applied to each dataset were adjusted based on the result of running the model selection program on MEGA X^57^ (Supplementary table S6). We also performed maximum-likelihood phylogenetic analysis using PhyML version 3.1_1 supplied on NGPhylogeny.fr homepage (https://ngphylogeny.fr/). Model selection program, ProtTest version 3.4.2^58^ was performed to set the evolutionary model, the proportion of invariant sites and the gamma distribution parameter on each dataset (Supplementary table S6), while other parameters remained default. In this study, we applied BIC (Bayesian Information Criterion) to the model selection in maximum-likelihood phylogenetic analyses. Reliability of maximum-likelihood trees was examined by bootstrap analysis based on 1000 replicates. Polychotomies were generated by collapsing the nodes with a bootstrap value lower than 50. We mainly discuss the nodes that remained in the polychotomous trees.

### Phylogenetic analyses of CB, VWA, and Laminin G domains in molluscan shell matrix proteins

In order to reconstruct evolutionary relationships among shell-specific VWA-CB dcps, phylogenetic analyses were performed on 84 and 187 alignable amino acid residues of CB and VWA domains, respectively. Domains of the VWA-CB dcp of the brachiopod, *Lingula anatina*, were taken as outgroups. Predicted domain structures of proteins and places where SMPs were identified are illustrated in Fig. 1a. Phylogenetic analyses were also performed on the Laminin G domain, which was identified by SMART and BLAST domain searches, for 210 amino acid residues and on the concatenated CB and Laminin G domains for 282 amino acid residues, respectively. The CB, VWA and Laminin G domains of Lan_1530^42^, a VWA-CB dcp of *L. anatina*, was used as the outgroup.

### Phylogenetic analyses on chitobiases

A combined domain search with SMART and BLAST (E-value: 10^−5^) was performed on CGI_10007856, Pfu_20027 and two adult shell-chitobiases CGI_10007857 and Pfu_20028^13,39^, revealing that they all possess conserved sequences of the four domains, CHB_HEX domain (IPR004866), Glyco_hydro_20b domain (IPR015882), Glyco_hydro_20 domain (IPR015883) and CHB_HEX_C domain (IPR004867) (Fig. 4a). Phylogenetic analyses on chitobiases were performed using genes possessing all four domains via combined domain searches in the four mollusc species, *C. gigas*, *P. fucata*, *L. gigantea*, and *O. bimaculoides* (Supplementary table S3). Shell-specific chitobiases are indicated by blue (*C. gigas*), orange (*P. fucata*) and green (*L. gigantea*) arrowheads. A sequence of the brachiopod, *Lingula anatina*, Lan_013407630^42^, containing the same four domains was taken as the outgroup. Trees were generated based on 1066 amino acid residues.

### Phylogenetic analyses of carbonic anhydrase (CA) in molluscs

The CA domain is another common domain identified in both larval and adult shell proteomes of *C. gigas* and *P. fucata* in our previous study^13^. CA genes are highly expanded in molluscs compared with other protostomes^13^, though reports of shell-specific CAs are still sparse and none has been reported from cephalopods. In order to determine the origin of shell-specific CAs in molluscs, phylogenetic analyses were performed on the CA domains of four molluscs, *Crassostrea gigas*, *Pinctada fucata*, *Lottia gigantea*, and *Octopus bimaculoides*, genomic and/or proteomic data of which reside in public databases (Supplementary table S5). Trees were generated based on 935 amino acid residues. A CA domain of the plant, *Arabidopsis thaliana*, was used as the outgroup*.* Shell-specific CAs are indicated by blue (*C. gigas*), orange (*P. fucata*) and green (*L. gigantea*) arrowheads. Phylogenetic analyses were also performed on the 494 aligned amino acid residues of CAs of molluscan shells and those of humans in order to infer relationships among them.

## Conclusion

We scrutinized the evolutionary histories of several SMPs that may be important for formation of both larval and adult shells, and using proteomic, genomic and transcriptomic data. We inferred that VWA-CB dcps and BMSP, as well as chitobiase, were already present in larval and adult shells of the common ancestor of bivalves before the speciation of *C. gigas* and *P. fucata*. On the other hand, in carbonic anhydrase SMPs, common SMPs that expanded widely among molluscs, the gene duplications that gave rise to separate deployments of larval and adult SMPs are inferred to have occurred after divergence of those two bivalves, which is more recent than previously expected. However, origins and evolutionary scenarios may be more complicated than have been shown in this study. Systematic sampling of both larval and adult SMPs from more molluscan species and even across taxa should be considered in the future.

## Supplementary Information

Supplementary Figures.

Supplementary Tables.
