# Supplementary material for: Phylogenetic comparisons reveal mosaic histories of larval and adult shell matrix protein deployment in pteriomorph bivalves
Source: Sci Rep. 2020 Dec 17;10:22140. doi: 10.1038/s41598-020-79330-x (PMC7747718; doi:10.1038/s41598-020-79330-x)
Supplement: Supplementary file 1 — Supplementary Figures. [file 41598_2020_79330_MOESM1_ESM.pdf]

Article (Discoveries)

Phylogenetic comparisons reveal mosaic histories of larval and adult shell matrix protein deployment in pteriomorph bivalves

Ran Zhao[1,8,*], Takeshi Takeuchi[2], Ryo Koyanagi[3], Alejandro Villar-Briones[4], Lixy Yamada[5], Hitoshi Sawada[5], Akito Ishikawa[1], Shunsuke Iwanaga[6], Kiyohito Nagai[7], Yuqi Che[8], Noriyuki Satoh[2] and Kazuyoshi Endo[1]

[1] Department of Earth and Planetary Science, Graduate School of Science, University of Tokyo, Bunkyo-ku, Tokyo 113-0033, Japan

[2] Marine Genomics Unit, Okinawa Institute of Science and Technology Graduate University, Onna, Okinawa, 904-0495 Japan

[3] DNA Sequencing Section, Okinawa Institute of Science and Technology Graduate University, Onna, Okinawa, 904-0495 Japan

[4] Instrumental Analysis Section, Okinawa Institute of Science and Technology Graduate University, Onna, Okinawa, 904-0495 Japan

[5] Sugashima Marine Biological Laboratory, Graduate School of Science, Nagoya University, Sugashima, Toba 517-0004, Japan

[6] Nagasaki Prefectural Institute of Fisheries, Nagasaki, Nagasaki 851-2213, Japan

[7] Pearl Research Institute, Mikimoto CO., LTD, Shima, Mie 517-0403, Japan

[8] Department of Biology, Shenzhen MSU-BIT University, 1 International University Park Road, Dayun New Town, Longgang District, Shenzhen, Guangdong Province, P.R. China

[*] Correspondence: zhao_ran13@hotmail.com (Ran. Zhao)

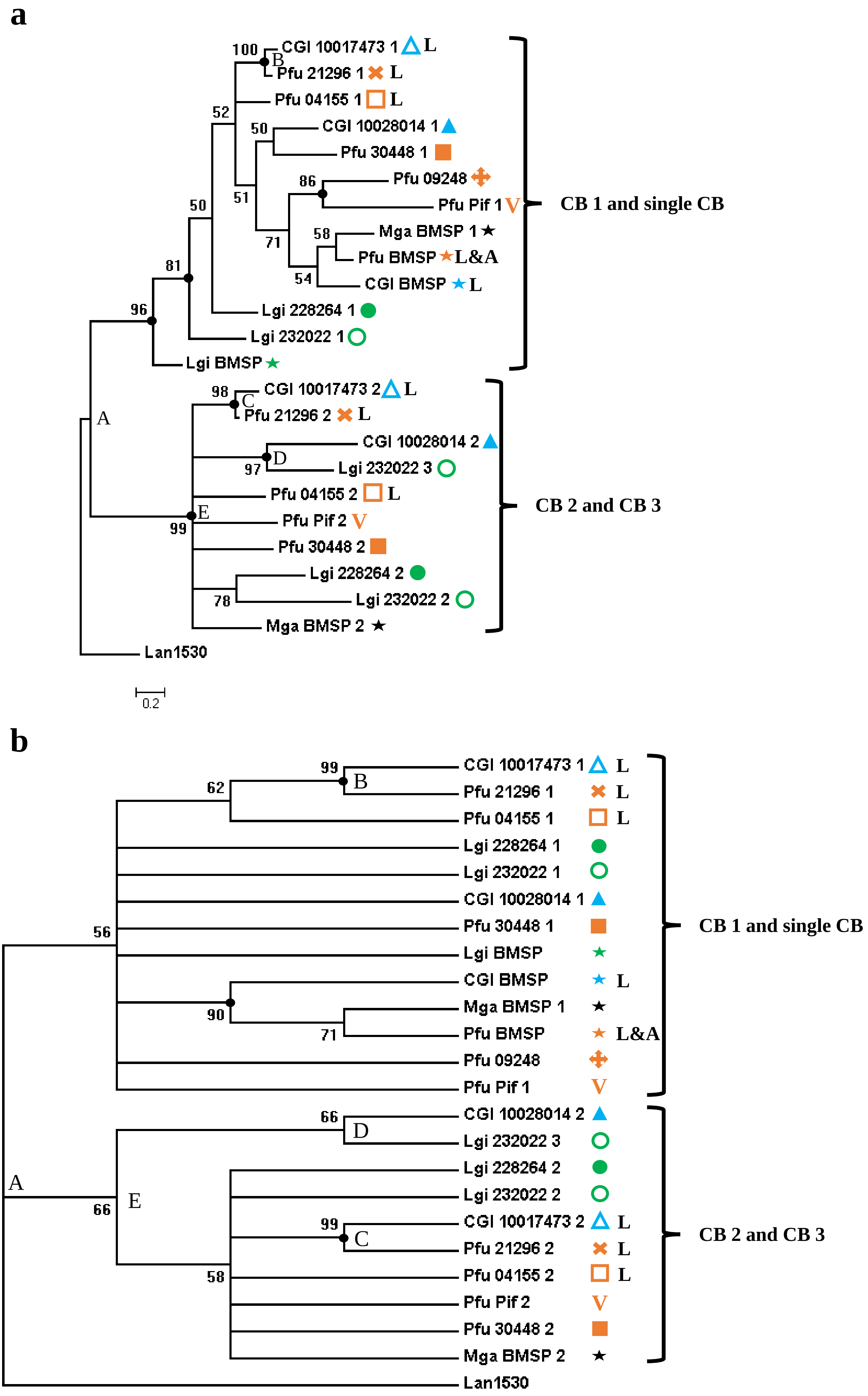
a
CGI 10017473 1 L
Pfu 21296 1 L
Pfu 04155 1 L
CGI 10028014 1
Pfu 30448 1
Pfu 09248
Pfu Pif 1 V
Mga BMSP 1
Pfu BMSP L&A
CGI BMSP L
Lgi 228264 1
Lgi 232022 1
Lgi BMSP
CGI 10017473 2 L
Pfu 21296 2 L
CGI 10028014 2
Lgi 232022 3
Pfu 04155 2 L
Pfu Pif 2 V
Pfu 30448 2
Lgi 228264 2
Lgi 232022 2
Mga BMSP 2
Lan1530
100
B
52
50
86
51
71
58
54
50
81
96
A
98
C
97
D
E
99
78
CB 1 and single CB
CB 2 and CB 3
0.2
b
CGI 10017473 1 L
Pfu 21296 1 L
Pfu 04155 1 L
Lgi 228264 1
Lgi 232022 1
CGI 10028014 1
Pfu 30448 1
Lgi BMSP
CGI BMSP L
Mga BMSP 1
Pfu BMSP L&A
Pfu 09248
Pfu Pif 1 V
CGI 10028014 2
Lgi 232022 3
Lgi 228264 2
Lgi 232022 2
CGI 10017473 2 L
Pfu 21296 2 L
Pfu 04155 2 L
Pfu Pif 2 V
Pfu 30448 2
Mga BMSP 2
Lan1530
99
B
62
56
90
71
66
D
A
66
E
99
C
58
CB 1 and single CB
CB 2 and CB 3

c

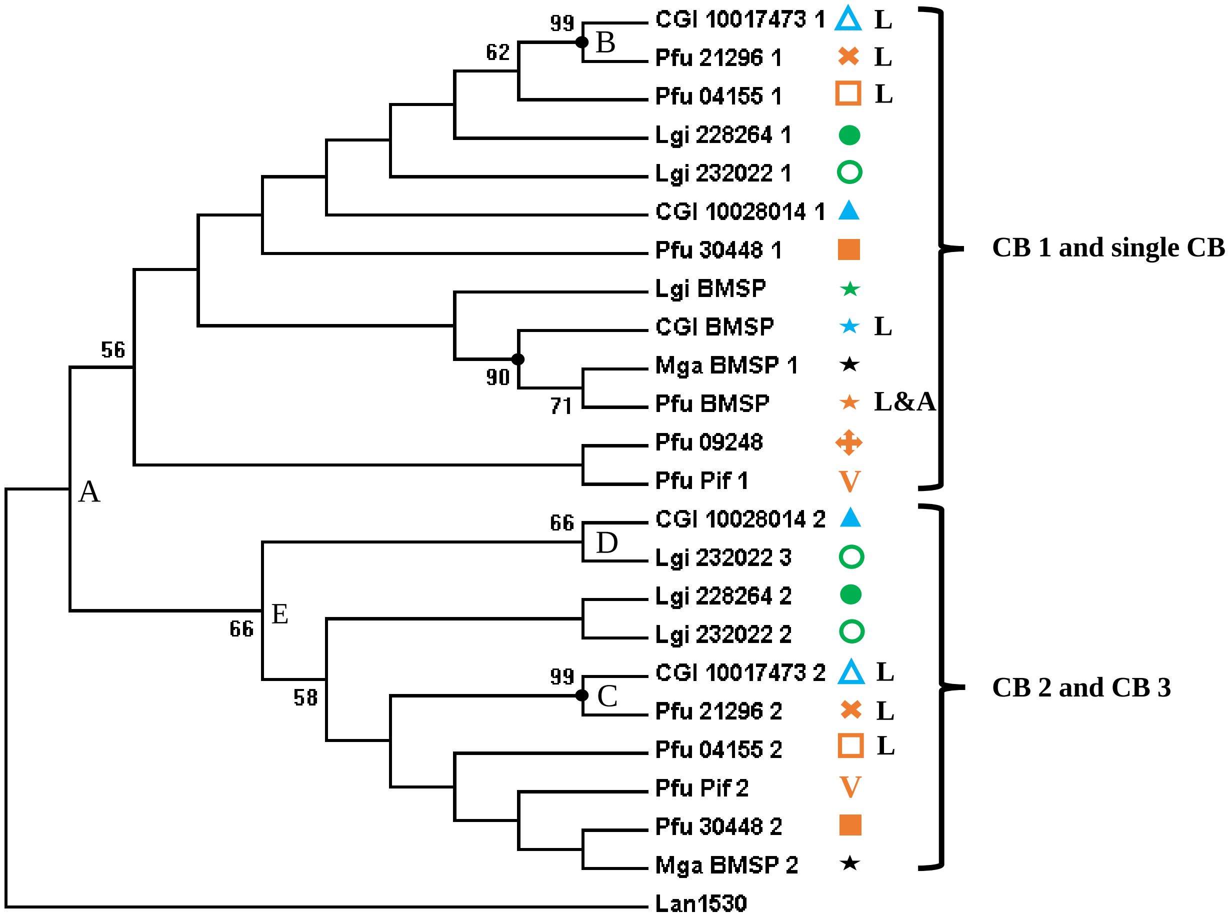
99
62
B
CGI 10017473 1 L
Pfu 21296 1 L
Pfu 04155 1 L
Lgi 228264 1
Lgi 232022 1
CGI 10028014 1
Pfu 30448 1
Lgi BMSP
CGI BMSP L
Mga BMSP 1
Pfu BMSP L&A
90
71
56
Pfu 09248
Pfu Pif 1 V
A
CB 1 and single CB
66
D
CGI 10028014 2
Lgi 232022 3
E
66
Lgi 228264 2
Lgi 232022 2
99
C
CGI 10017473 2 L
Pfu 21296 2 L
58
Pfu 04155 2 L
Pfu Pif 2 V
Pfu 30448 2
Mga BMSP 2
CB 2 and CB 3
Lan1530

d

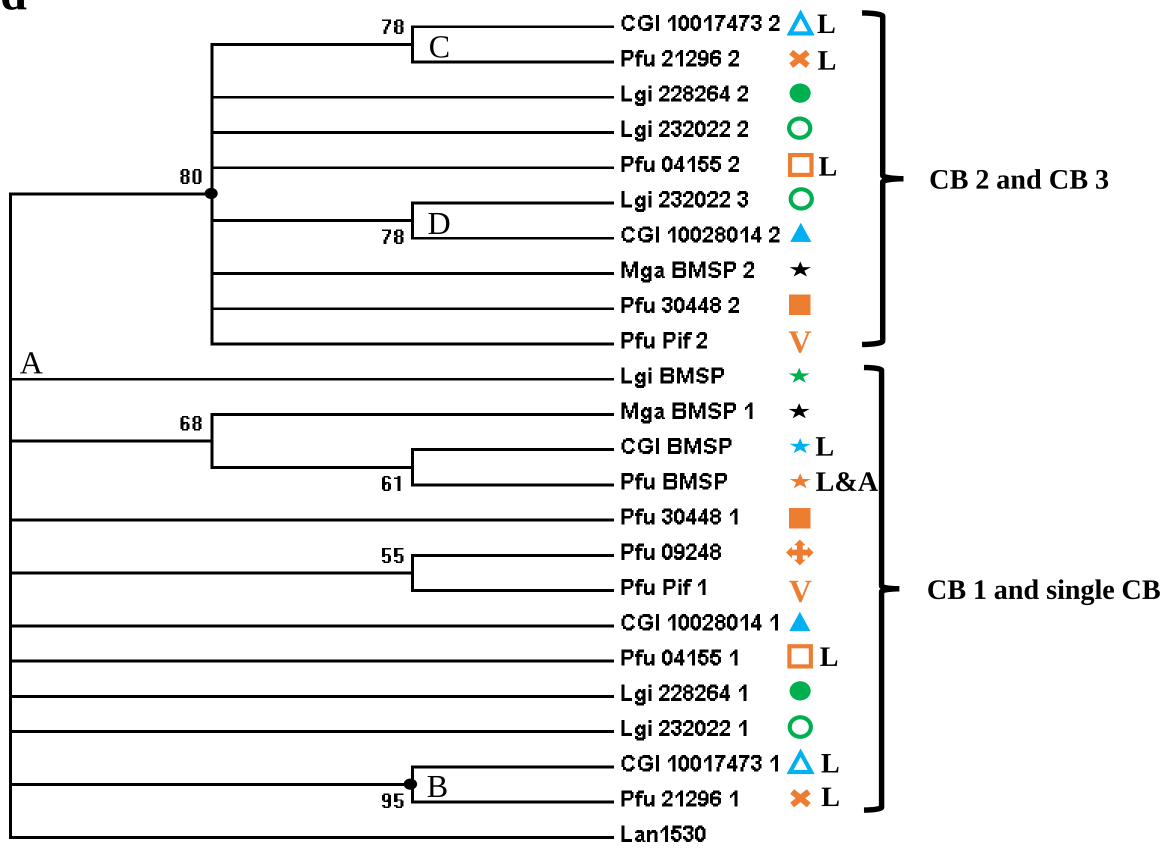
78
C
CGI 10017473 2 L
Pfu 21296 2 L
Lgi 228264 2
Lgi 232022 2
80
Pfu 04155 2 L
Lgi 232022 3
D
78
CGI 10028014 2
Mga BMSP 2
Pfu 30448 2
Pfu Pif 2 V
CB 2 and CB 3
A
Lgi BMSP
68
Mga BMSP 1
CGI BMSP L
61
Pfu BMSP L&A
Pfu 30448 1
55
Pfu 09248
Pfu Pif 1 V
CB 1 and single CB
CGI 10028014 1
Pfu 04155 1 L
Lgi 228264 1
Lgi 232022 1
CGI 10017473 1 L
B
95
Pfu 21296 1 L
Lan1530

e

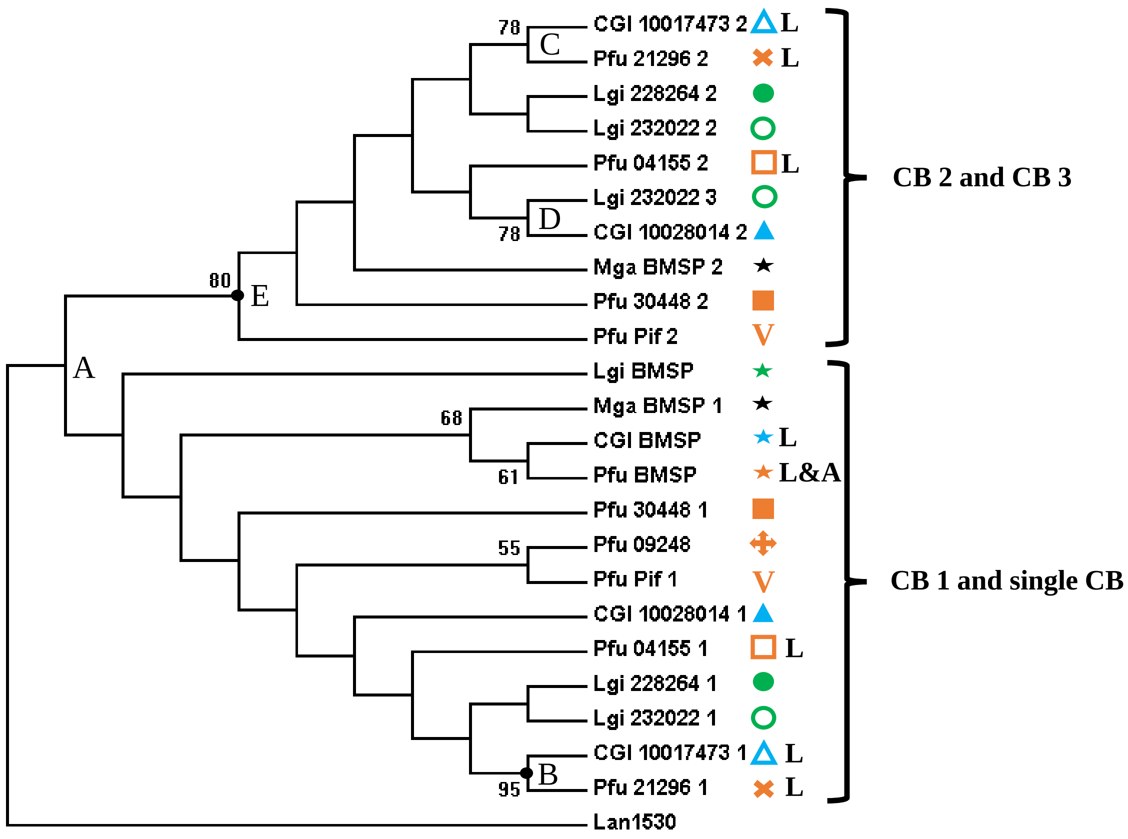

Supplementary Fig. 1 Phylogenetic analyses of CB domains of VWA-CB dcps of molluscan shells based on alignment of 84 amino acid residues performed by MrBayes (**a**), MEGA X (**b, c**) and PhyML (**d**, **e**), respectively. **a** Phylogram of CB domains of VWA-CB dcps. The *bar* represents 0.2 substitutions per site. Polychotomy is generated if the posterior probability value of the node is <50. **b** Polychotomy is generated if the bootstrap value of the node is <50. **c** Original tree retaining dichotomies. **d** Polychotomy is generated if the bootstrap value of the node is <50. **e** Original tree retaining dichotomies. Posterior probability/Bootstrap values are shown if ≥50, and marked with black dots if ≥80. The same symbol marks as in Figure 1 are used to denote different SMPs. Larval SMPs are marked by "L". Domains of Pfu_BMSP identified from both larval and adult shells of *P. fucata* are indicated by "L&A". Dp, duplication. CGI, *Crassostrea gigas*; Pfu, *Pinctada fucata*; Mga, *Mytilus galloprovincialis*; Lgi, *Lottia gigantea*; Lan, *Lingula anatina*.

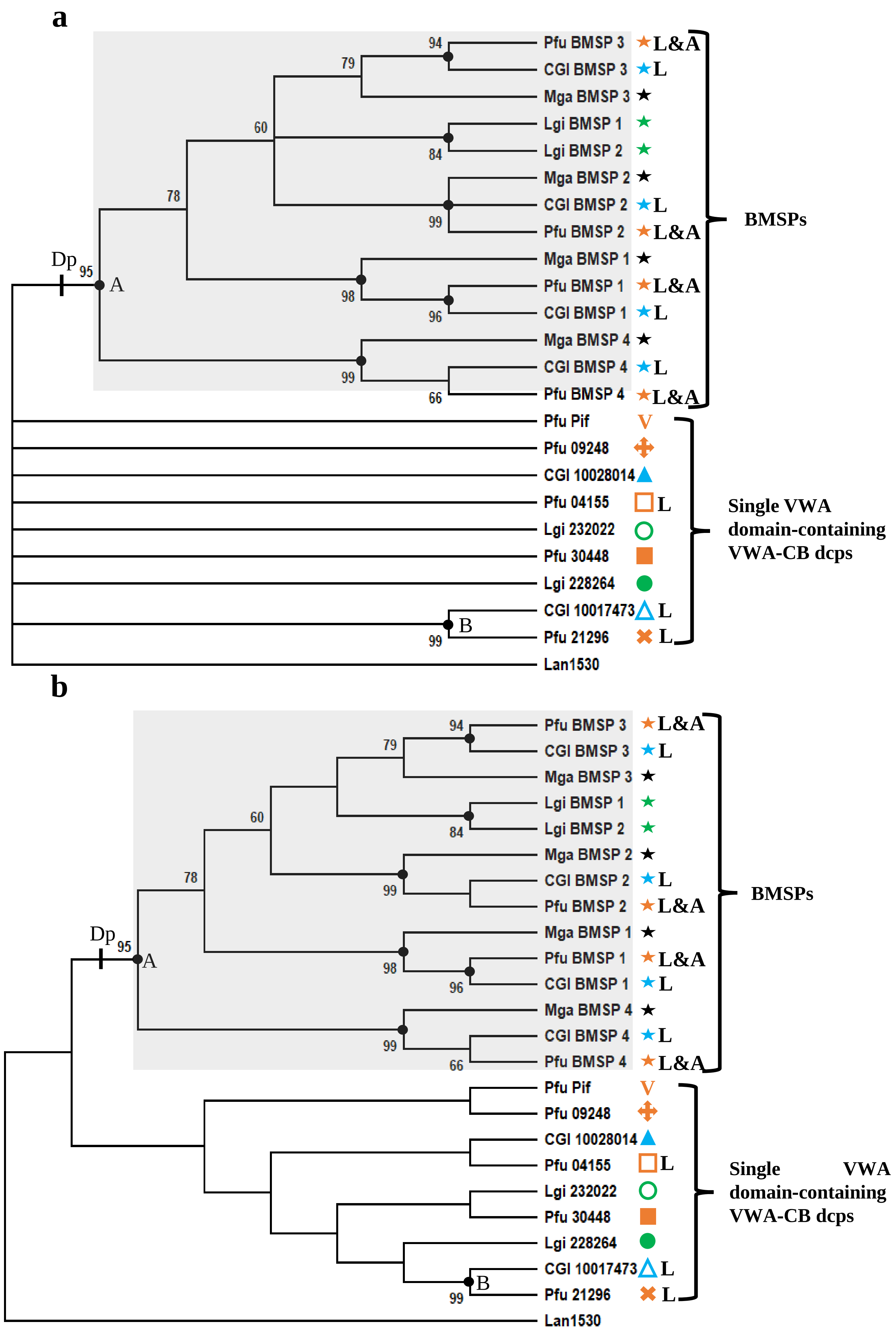

a
Dp
95
A
94
79
60
78
84
99
98
96
66
Pfu BMSP 3 L&A
CGI BMSP 3 L
Mga BMSP 3
Lgi BMSP 1
Lgi BMSP 2
Mga BMSP 2
CGI BMSP 2 L
Pfu BMSP 2 L&A
Mga BMSP 1
Pfu BMSP 1 L&A
CGI BMSP 1 L
Mga BMSP 4
CGI BMSP 4 L
Pfu BMSP 4 L&A
BMSPs
Pfu Pif V
Pfu 09248
CGI 10028014
Pfu 04155 L
Lgi 232022
Pfu 30448
Lgi 228264
CGI 10017473 L
Pfu 21296 L
B
Lan1530
Single VWA
domain-containing
VWA-CB dcps
b
Dp
95
A
B
BMSPs
Single VWA
domain-containing
VWA-CB dcps
Lan1530

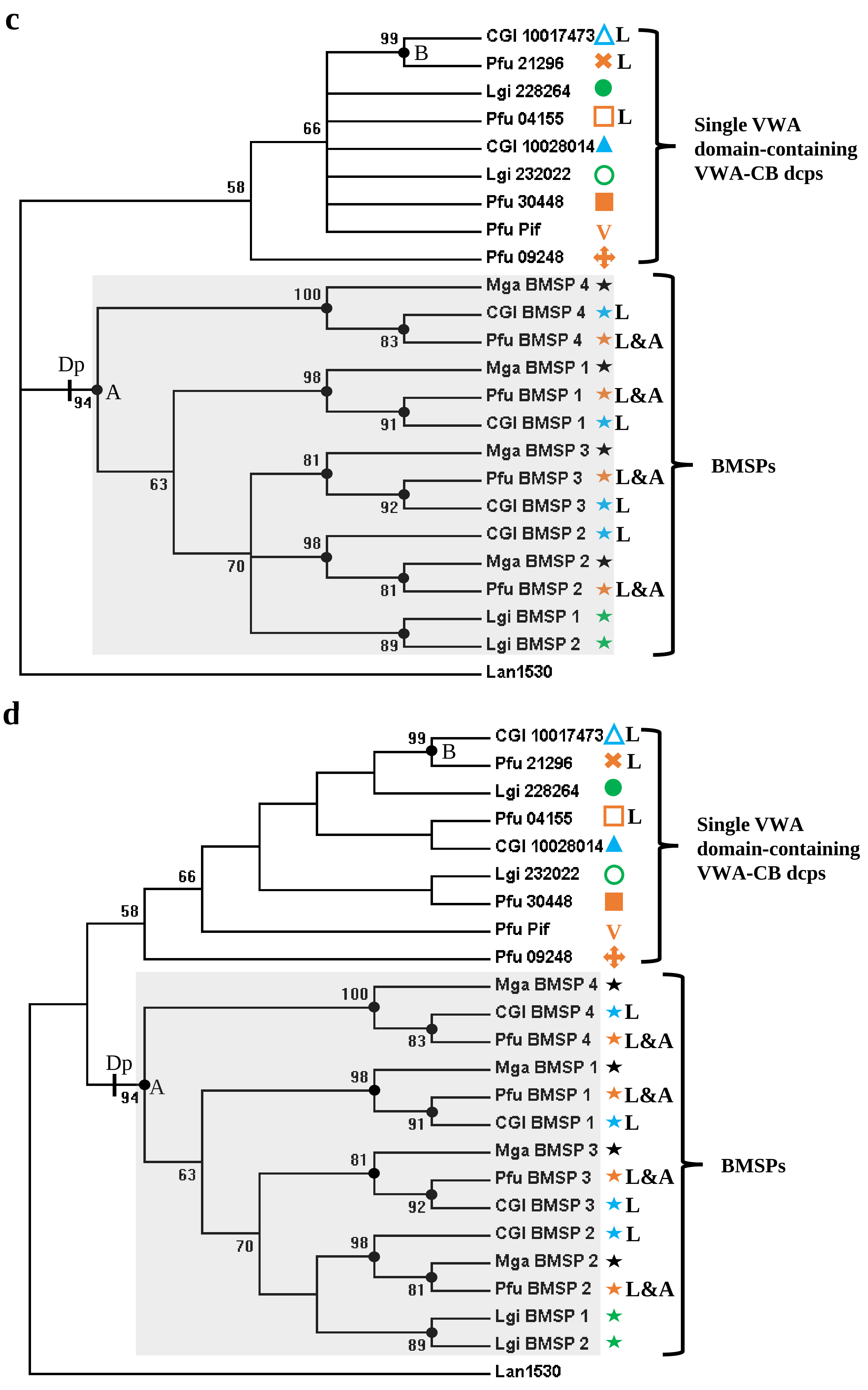
c
CGI 10017473 L
Pfu 21296 L
Lgi 228264
Pfu 04155 L
CGI 10028014
Lgi 232022
Pfu 30448
Pfu Pif V
Pfu 09248
Mga BMSP 4
CGI BMSP 4 L
Pfu BMSP 4 L&A
Mga BMSP 1
Pfu BMSP 1 L&A
CGI BMSP 1 L
Mga BMSP 3
Pfu BMSP 3 L&A
CGI BMSP 3 L
CGI BMSP 2 L
Mga BMSP 2
Pfu BMSP 2 L&A
Lgi BMSP 1
Lgi BMSP 2
Lan1530
99
B
66
58
100
83
Dp
94
A
98
91
81
92
63
98
70
81
89
Single VWA domain-containing VWA-CB dcps
BMSPs
d
CGI 10017473 L
Pfu 21296 L
Lgi 228264
Pfu 04155 L
CGI 10028014
Lgi 232022
Pfu 30448
Pfu Pif V
Pfu 09248
Mga BMSP 4
CGI BMSP 4 L
Pfu BMSP 4 L&A
Mga BMSP 1
Pfu BMSP 1 L&A
CGI BMSP 1 L
Mga BMSP 3
Pfu BMSP 3 L&A
CGI BMSP 3 L
CGI BMSP 2 L
Mga BMSP 2
Pfu BMSP 2 L&A
Lgi BMSP 1
Lgi BMSP 2
Lan1530
99
B
66
58
100
83
Dp
94
A
98
91
81
92
63
98
70
81
89
Single VWA domain-containing VWA-CB dcps
BMSPs

e

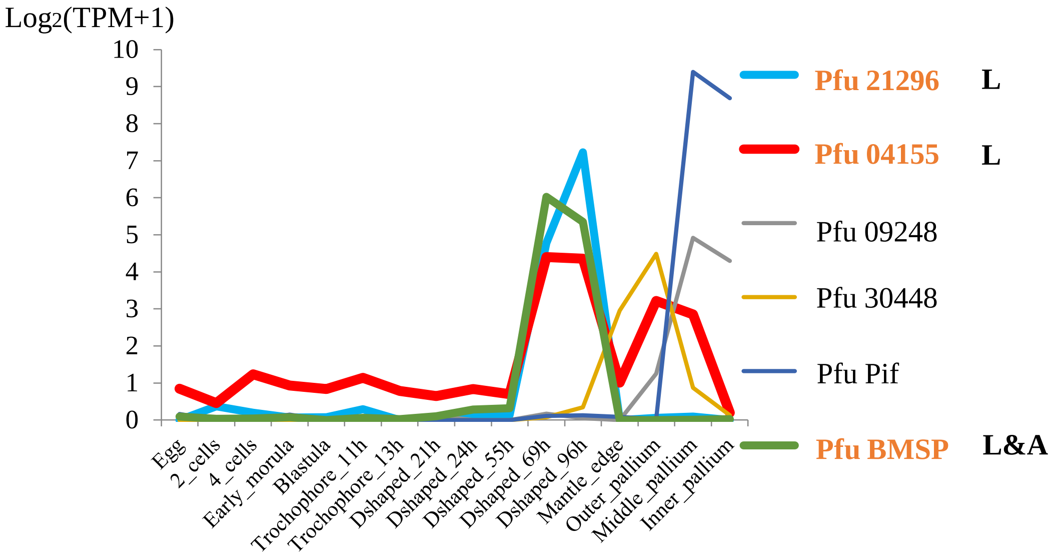

Supplementary Fig. 2 Maximum-likelihood phylogenetic analyses of VWA domains of VWA-CB dcps of molluscan shells based on alignment of 187 amino acid residues performed by MEGA X (**a**, **b**) and PhyML (**c**, **d**), respectively. **a** Polychotomy is generated if the bootstrap value of the node is <50. **b** Original tree retaining dichotomies. **c** Polychotomy is generated if the bootstrap value of the node is <50. **d** Original tree retaining dichotomies. Bootstrap values are shown if ≥50, and marked with black dots if ≥80. **e** Expression patterns of transcripts of VWA-CB dcps of *P. fucata*. Larval SMPs are indicated by bold lines and orange characters. The same symbol marks as in Figure 1 are used to denote different SMPs. Larval SMPs are marked by “L”. Domains of Pfu_BMSP identified from both larval and adult shells of *P. fucata* are indicated by “L&A”. Dp, duplication. CGI, *Crassostrea gigas*; Pfu, *Pinctada fucata*; Mga, *Mytilus galloprovincialis*; Lgi, *Lottia gigantea*; Lan, *Lingula anatina*.

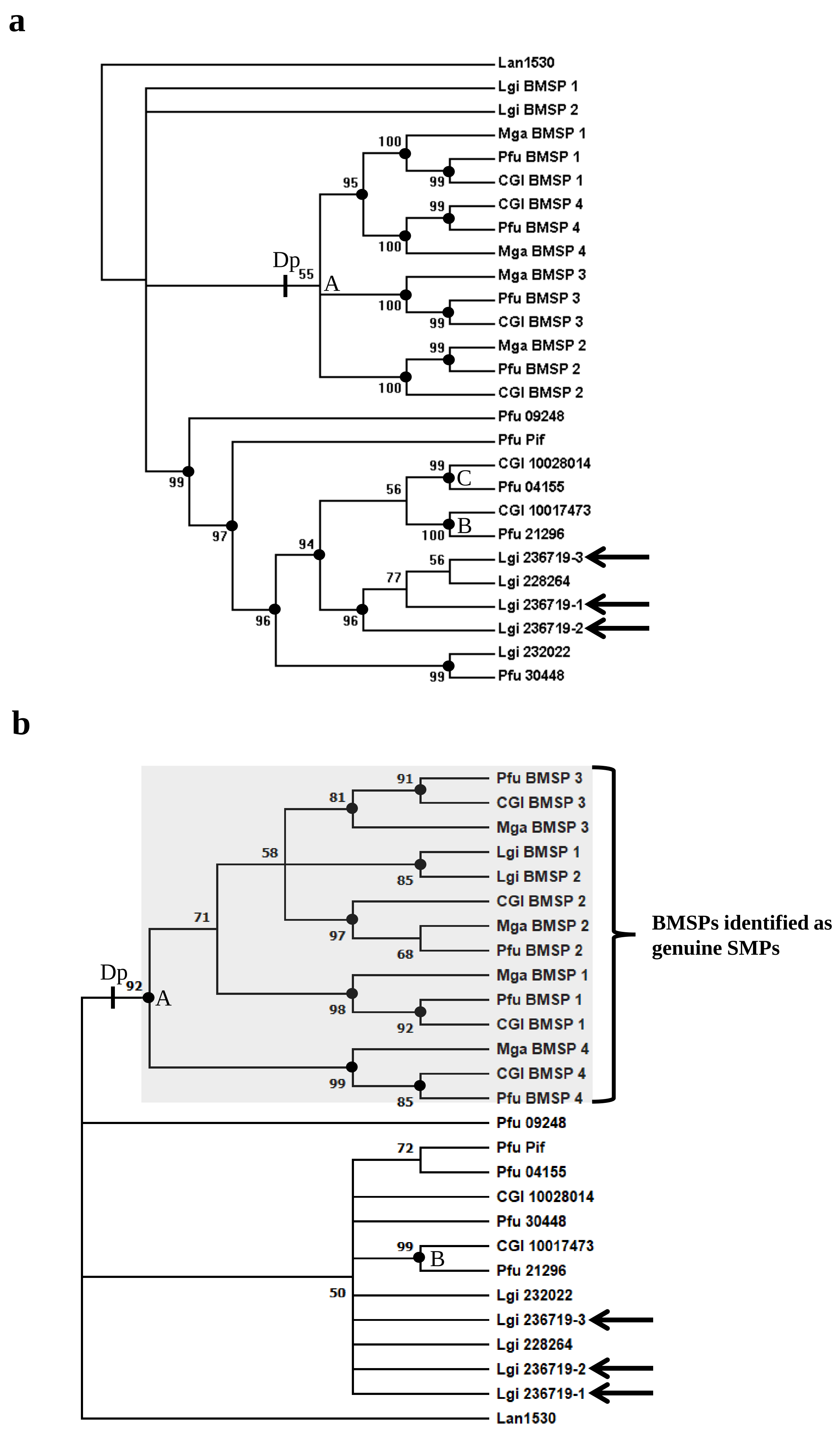
a
Lan1530
Lgi BMSP 1
Lgi BMSP 2
Mga BMSP 1
Pfu BMSP 1
CGI BMSP 1
CGI BMSP 4
Pfu BMSP 4
Mga BMSP 4
Mga BMSP 3
Pfu BMSP 3
CGI BMSP 3
Mga BMSP 2
Pfu BMSP 2
CGI BMSP 2
Pfu 09248
Pfu Pif
CGI 10028014
Pfu 04155
CGI 10017473
Pfu 21296
Lgi 236719-3
Lgi 228264
Lgi 236719-1
Lgi 236719-2
Lgi 232022
Pfu 30448
Dp55
A
B
C
b
Pfu BMSP 3
CGI BMSP 3
Mga BMSP 3
Lgi BMSP 1
Lgi BMSP 2
CGI BMSP 2
Mga BMSP 2
Pfu BMSP 2
Mga BMSP 1
Pfu BMSP 1
CGI BMSP 1
Mga BMSP 4
CGI BMSP 4
Pfu BMSP 4
Pfu 09248
Pfu Pif
Pfu 04155
CGI 10028014
Pfu 30448
CGI 10017473
Pfu 21296
Lgi 232022
Lgi 236719-3
Lgi 228264
Lgi 236719-2
Lgi 236719-1
Lan1530
Dp92
A
B
BMSPs identified as genuine SMPs

c

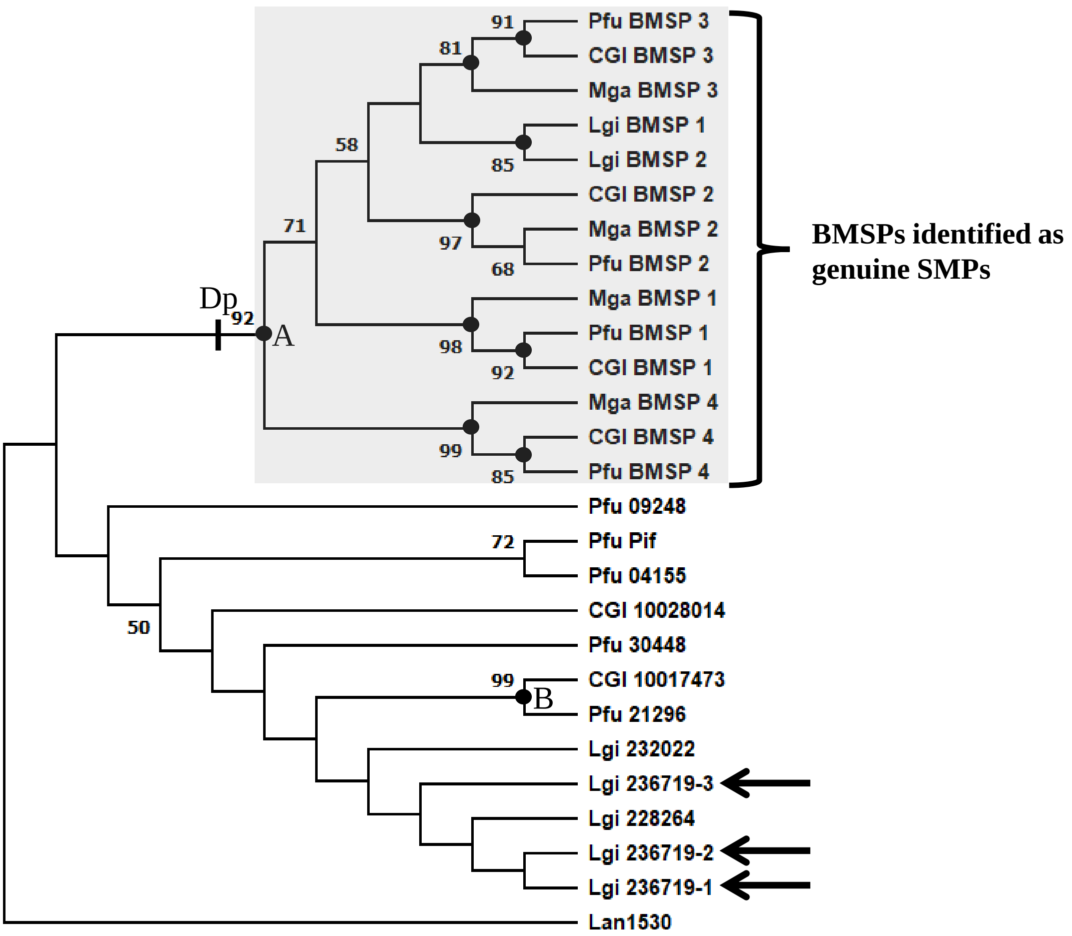
Dp
92
A
B
91
81
85
58
71
97
68
98
92
99
85
72
50
99
Pfu BMSP 3
CGI BMSP 3
Mga BMSP 3
Lgi BMSP 1
Lgi BMSP 2
CGI BMSP 2
Mga BMSP 2
Pfu BMSP 2
Mga BMSP 1
Pfu BMSP 1
CGI BMSP 1
Mga BMSP 4
CGI BMSP 4
Pfu BMSP 4
Pfu 09248
Pfu Pif
Pfu 04155
CGI 10028014
Pfu 30448
CGI 10017473
Pfu 21296
Lgi 232022
Lgi 236719-3
Lgi 228264
Lgi 236719-2
Lgi 236719-1
Lan1530
BMSPs identified as genuine SMPs

d

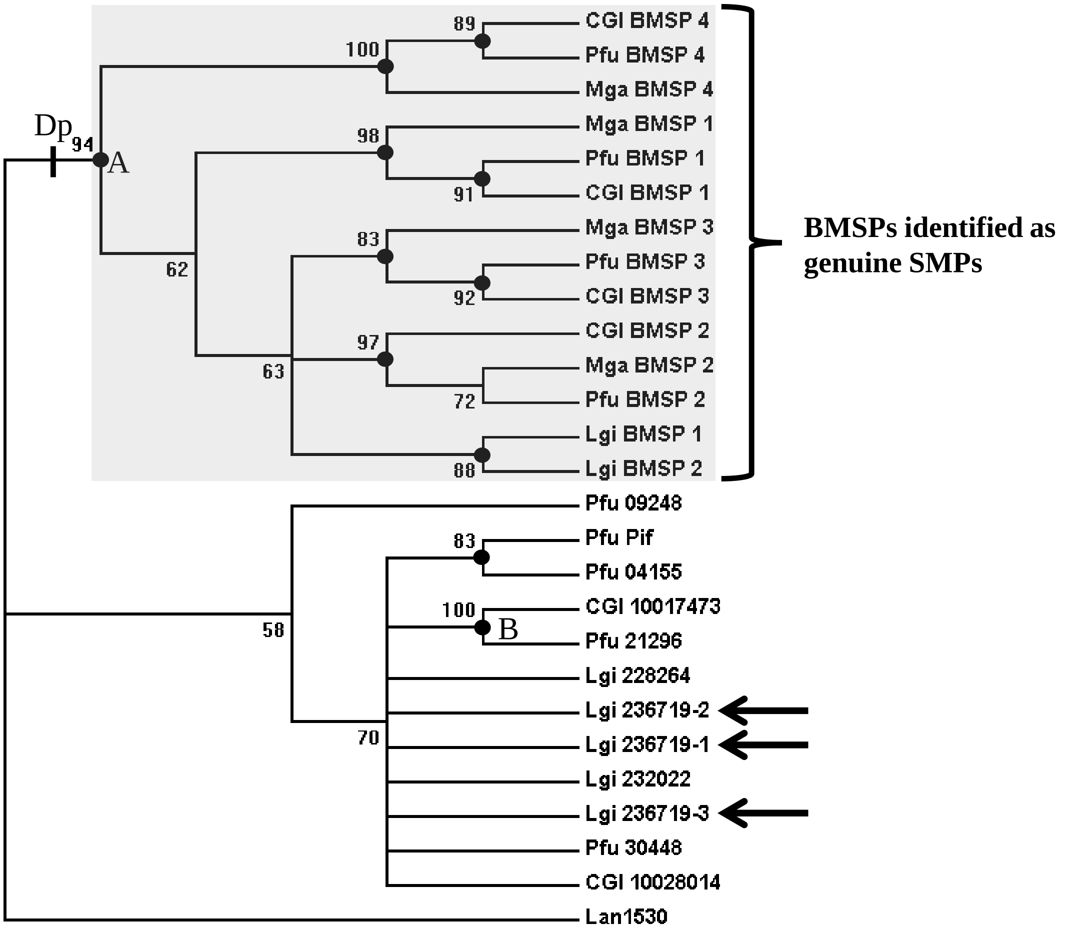
Dp
94
A
B
89
100
98
91
83
92
62
97
63
72
88
83
100
58
70
CGI BMSP 4
Pfu BMSP 4
Mga BMSP 4
Mga BMSP 1
Pfu BMSP 1
CGI BMSP 1
Mga BMSP 3
Pfu BMSP 3
CGI BMSP 3
CGI BMSP 2
Mga BMSP 2
Pfu BMSP 2
Lgi BMSP 1
Lgi BMSP 2
Pfu 09248
Pfu Pif
Pfu 04155
CGI 10017473
Pfu 21296
Lgi 228264
Lgi 236719-2
Lgi 236719-1
Lgi 232022
Lgi 236719-3
Pfu 30448
CGI 10028014
Lan1530
BMSPs identified as genuine SMPs

e

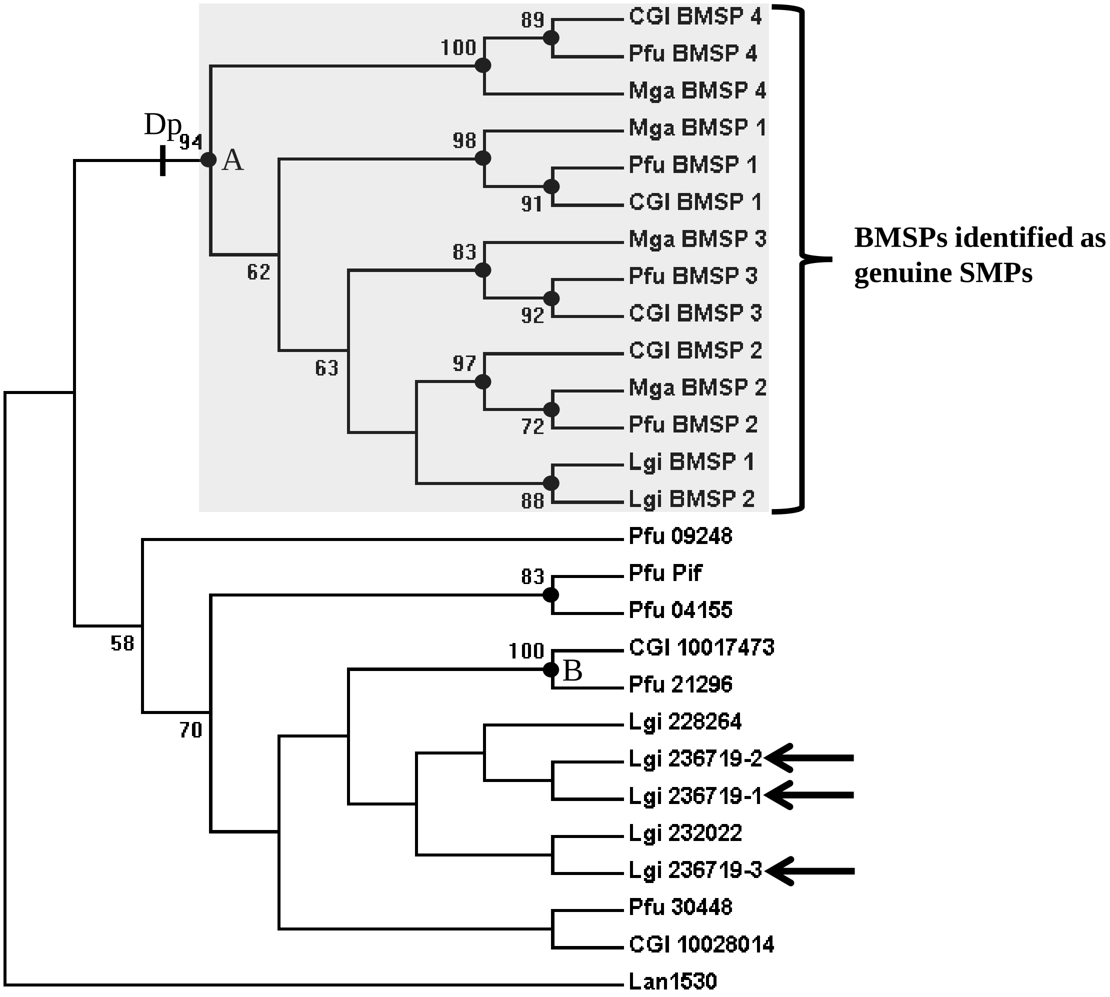

Supplementary Fig. 3 Phylogenetic analyses of VWA domains of VWA-CB dcps of molluscan shells and LG236719 on 187 amino acid residues performed by MrBayes (**a**), MEGA X (**b, c**) and PhyML (**d**, **e**), respectively. **a** Polychotomy is generated if the posterior probability value of the node is <50. **b** Polychotomy is generated if the bootstrap value of the node is <50. **c** Original tree retaining dichotomies. **d** Polychotomy is generated if the bootstrap value of the node is <50. **e** Original tree retaining dichotomies. Posterior probability/Bootstrap values are shown if ≥50, and marked with black dots if ≥80. VWA domains of LG236719 are indicated by arrowheads. The group formed by BMSPs which were identified as genuine SMPs are indicated. Dp, duplication. CGI, *Crassostrea gigas*; Pfu, *Pinctada fucata*; Mga, *Mytilus galloprovincialis*; Lgi, *Lottia gigantea*; Lan, *Lingula anatina*.

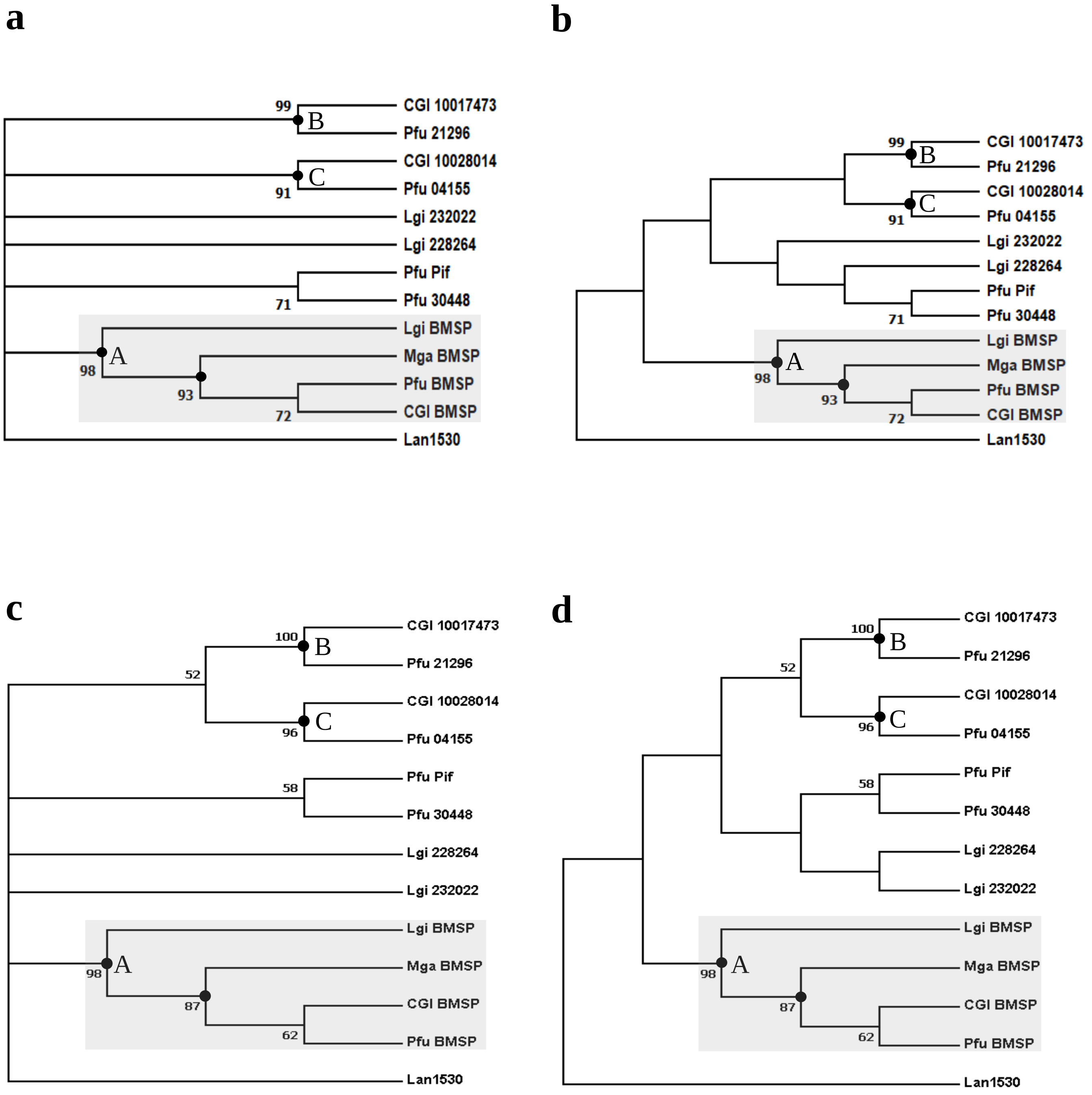

Supplementary Fig. 4 Maximum-likelihood phylogenetic analyses of Lamnin G domains of VWA-CB dcps of molluscan shells on 210 amino acid residues via MEGA X (**a, b**) and PhyML (**c**, **d**), respectively. **a** Polychotomy is generated if the bootstrap value of the node is <50. **b** Original tree retaining dichotomies. **c** Polychotomy is generated if the bootstrap value of the node is <50. **d** Original tree retaining dichotomies. Posterior probability/Bootstrap values are shown if ≥50, and marked with black dots if ≥80. CGI, *Crassostrea gigas*; Pfu, *Pinctada fucata*; Mga, *Mytilus galloprovincialis*; Lgi, *Lottia gigantea*; Lan, *Lingula anatina*.

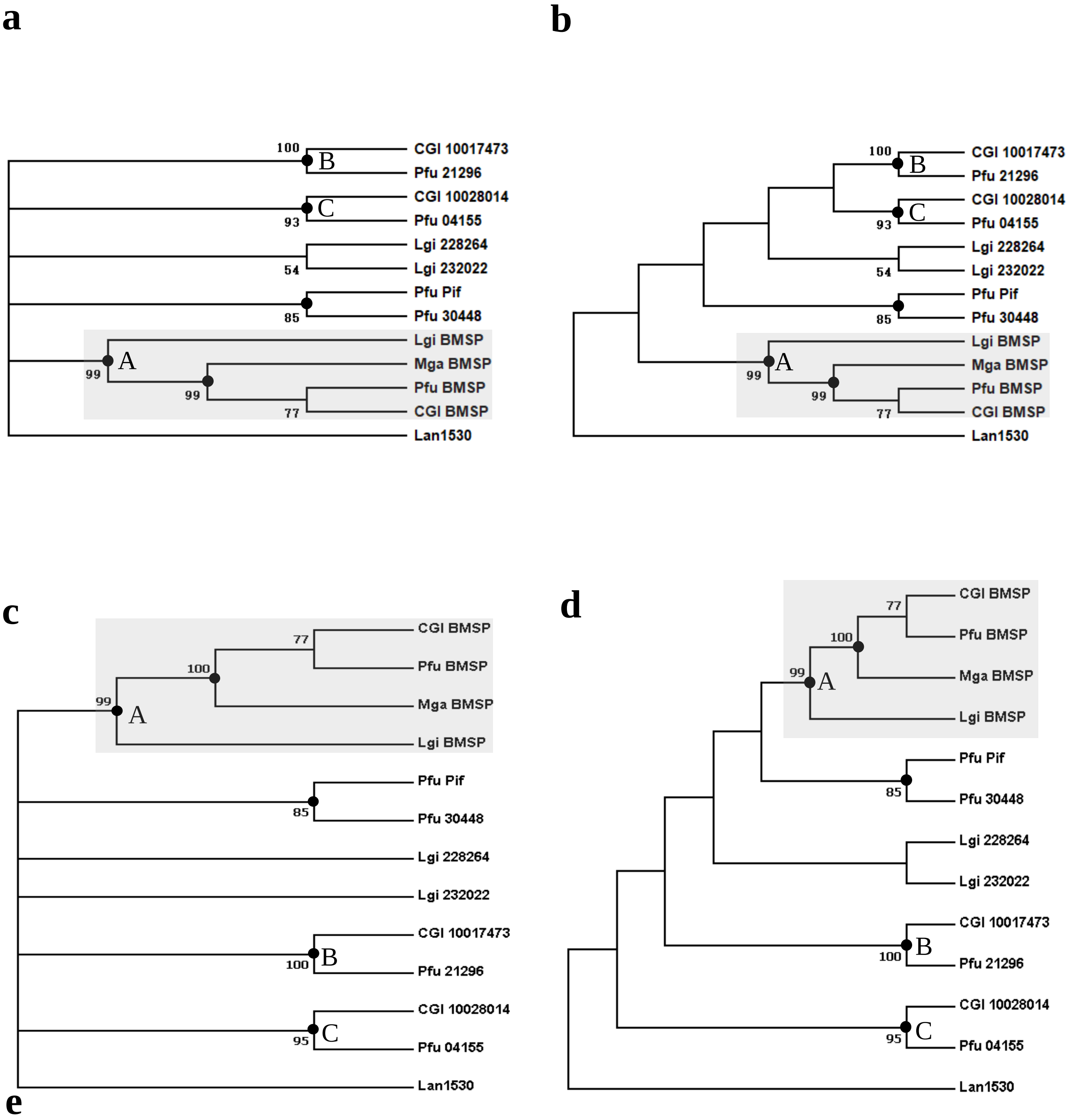

Supplementary Fig. 5 Maximum-likelihood phylogenetic analyses of concatenated sequences of CB and Laminin G domains of VWA-CB dcps of molluscan shells on 282 amino acid residues via MEGA X (**a, b**) and PhyML (**c**, **d**), respectively. **a** Polychotomy is generated if the bootstrap value of the node is <50. **b** Original tree retaining dichotomies. **c** Polychotomy is generated if the bootstrap value of the node is <50. **d** Original tree retaining dichotomies. Posterior probability/Bootstrap values are shown if ≥50, and marked with black dots if ≥80. CGI, *Crassostrea gigas*; Pfu, *Pinctada fucata*; Mga, *Mytilus galloprovincialis*; Lgi, *Lottia gigantea*; Lan, *Lingula anatina*.

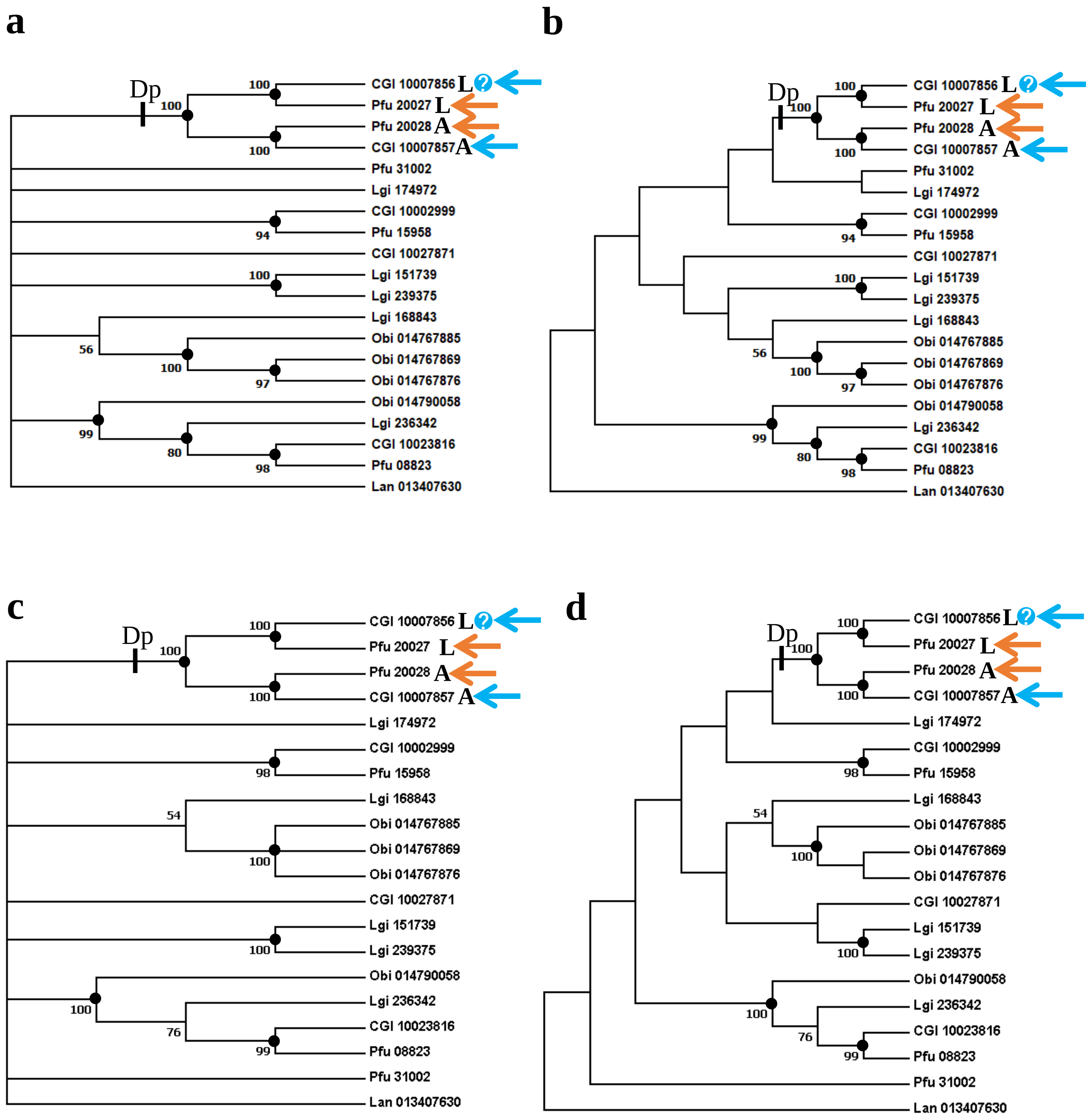

Supplementary Fig.6 Maximum-likelihood phylogenetic trees of chitobiases towards concatenated sequences of CHB_HEX domain (IPR004866), Glyco_hydro_20b domain (IPR015882), Glyco_hydro_20 domain (IPR015883) and CHB_HEX_C domain (IPR004867) on 1066 amino acid residues via MEGA 7 (**a**, **b**) and PhyML (**c**, **d**). **a** Polychotomy is generated if the bootstrap value of the node is <50. **b** Original tree retaining dichotomies. **c** Polychotomy is generated if the bootstrap value of the node is <50. **d** Original tree retaining dichotomies. SMPs are indicated by blue (*C. gigas*) and orange (*P. fucata*) arrowheads. The question mark indicates that whether or not the gene is encoding an SMP is uncertain. Bootstrap values are shown if ≥50, and marked with black dots if ≥80. Larval and adult SMPs are marked by "L" and "A", respectively. Dp, duplication. Cgi, *Crassostrea gigas*; Pfu, *Pinctada fucata*; Lgi, *Lottia gigantea*; Obi, *Octopus bimaculoides*. Lan, *Lingular anatina*.

a

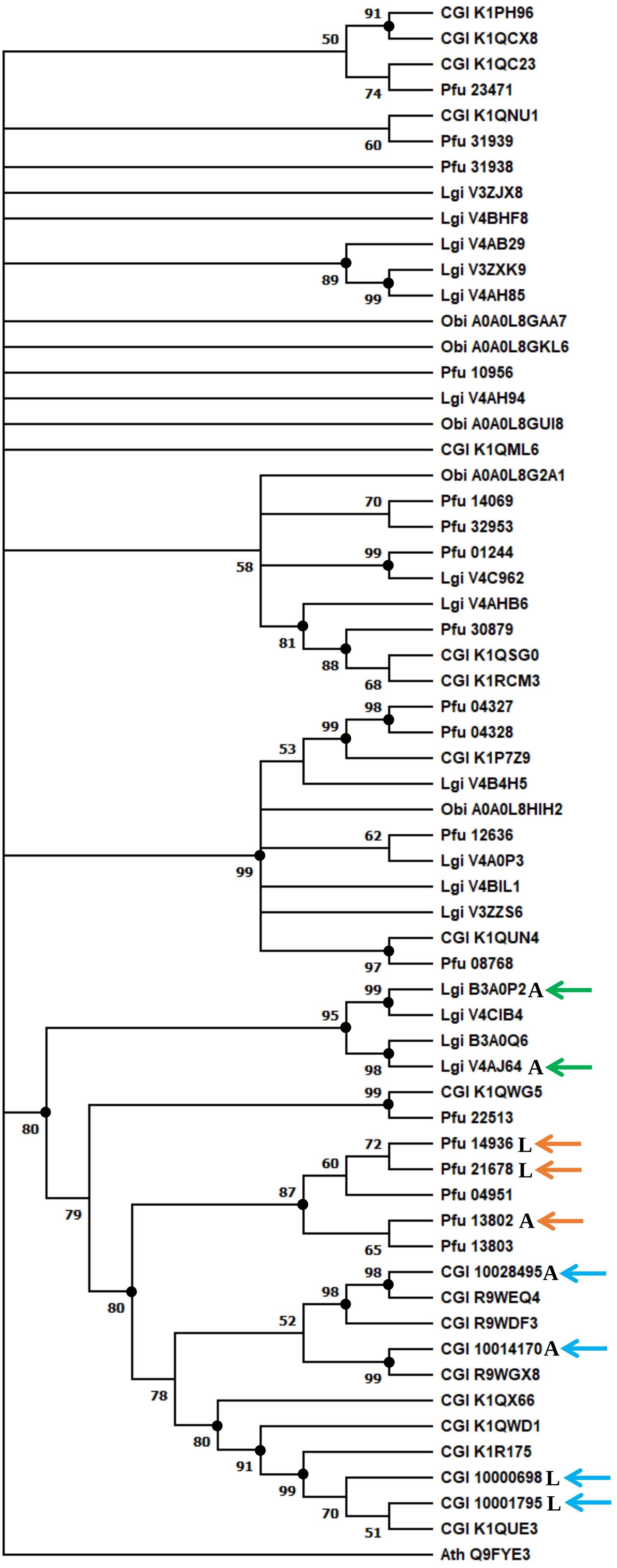

CGI K1PH96
CGI K1QCX8
CGI K1QC23
Pfu 23471
CGI K1QNU1
Pfu 31939
Pfu 31938
Lgi V3ZJX8
Lgi V4BHF8
Lgi V4AB29
Lgi V3ZXK9
Lgi V4AH85
Obi A0A0L8GAA7
Obi A0A0L8GKL6
Pfu 10956
Lgi V4AH94
Obi A0A0L8GUI8
CGI K1QML6
Obi A0A0L8G2A1
Pfu 14069
Pfu 32953
Pfu 01244
Lgi V4C962
Lgi V4AHB6
Pfu 30879
CGI K1QSG0
CGI K1RCM3
Pfu 04327
Pfu 04328
CGI K1P7Z9
Lgi V4B4H5
Obi A0A0L8HIH2
Pfu 12636
Lgi V4A0P3
Lgi V4BIL1
Lgi V3ZZS6
CGI K1QUN4
Pfu 08768
Lgi B3A0P2 A
Lgi V4CIB4
Lgi B3A0Q6
Lgi V4AJ64 A
CGI K1QWG5
Pfu 22513
Pfu 14936 L
Pfu 21678 L
Pfu 04951
Pfu 13802 A
Pfu 13803
CGI 10028495 A
CGI R9WEQ4
CGI R9WDF3
CGI 10014170 A
CGI R9WGX8
CGI K1QX66
CGI K1QWD1
CGI K1R175
CGI 10000698 L
CGI 10001795 L
CGI K1QUE3
Ath Q9FYE3
91
50
74
60
89
99
70
99
58
81
88
68
98
99
53
62
99
97
99
95
98
99
80
72
60
87
79
65
98
98
80
52
99
78
80
91
99
70
51

b

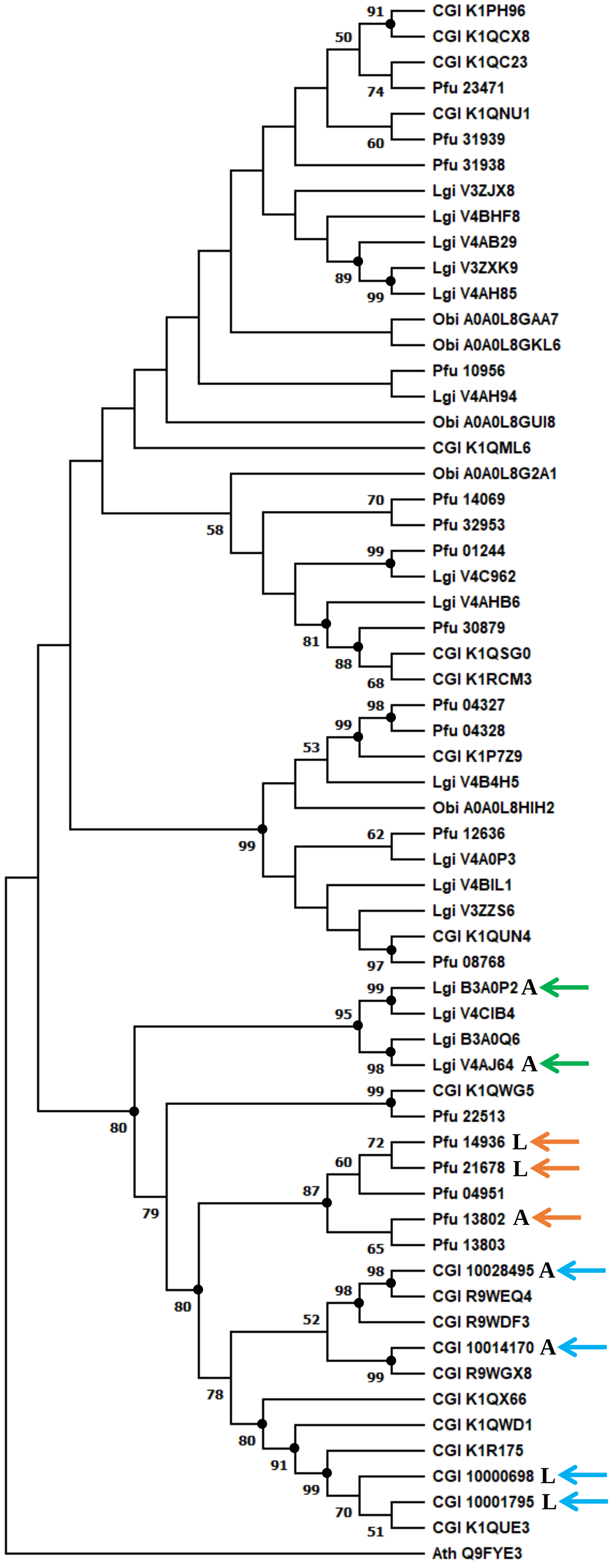
CGI K1PH96
CGI K1QCX8
CGI K1QC23
Pfu 23471
CGI K1QNU1
Pfu 31939
Pfu 31938
Lgi V3ZJX8
Lgi V4BHF8
Lgi V4AB29
Lgi V3ZXK9
Lgi V4AH85
Obi A0A0L8GAA7
Obi A0A0L8GKL6
Pfu 10956
Lgi V4AH94
Obi A0A0L8GUI8
CGI K1QML6
Obi A0A0L8G2A1
Pfu 14069
Pfu 32953
Pfu 01244
Lgi V4C962
Lgi V4AHB6
Pfu 30879
CGI K1QSG0
CGI K1RCM3
Pfu 04327
Pfu 04328
CGI K1P7Z9
Lgi V4B4H5
Obi A0A0L8HIH2
Pfu 12636
Lgi V4A0P3
Lgi V4BIL1
Lgi V3ZZS6
CGI K1QUN4
Pfu 08768
Lgi B3A0P2 A
Lgi V4CIB4
Lgi B3A0Q6
Lgi V4AJ64 A
CGI K1QWG5
Pfu 22513
Pfu 14936 L
Pfu 21678 L
Pfu 04951
Pfu 13802 A
Pfu 13803
CGI 10028495 A
CGI R9WEQ4
CGI R9WDF3
CGI 10014170 A
CGI R9WGX8
CGI K1QX66
CGI K1QWD1
CGI K1R175
CGI 10000698 L
CGI 10001795 L
CGI K1QUE3
Ath Q9FYE3

c

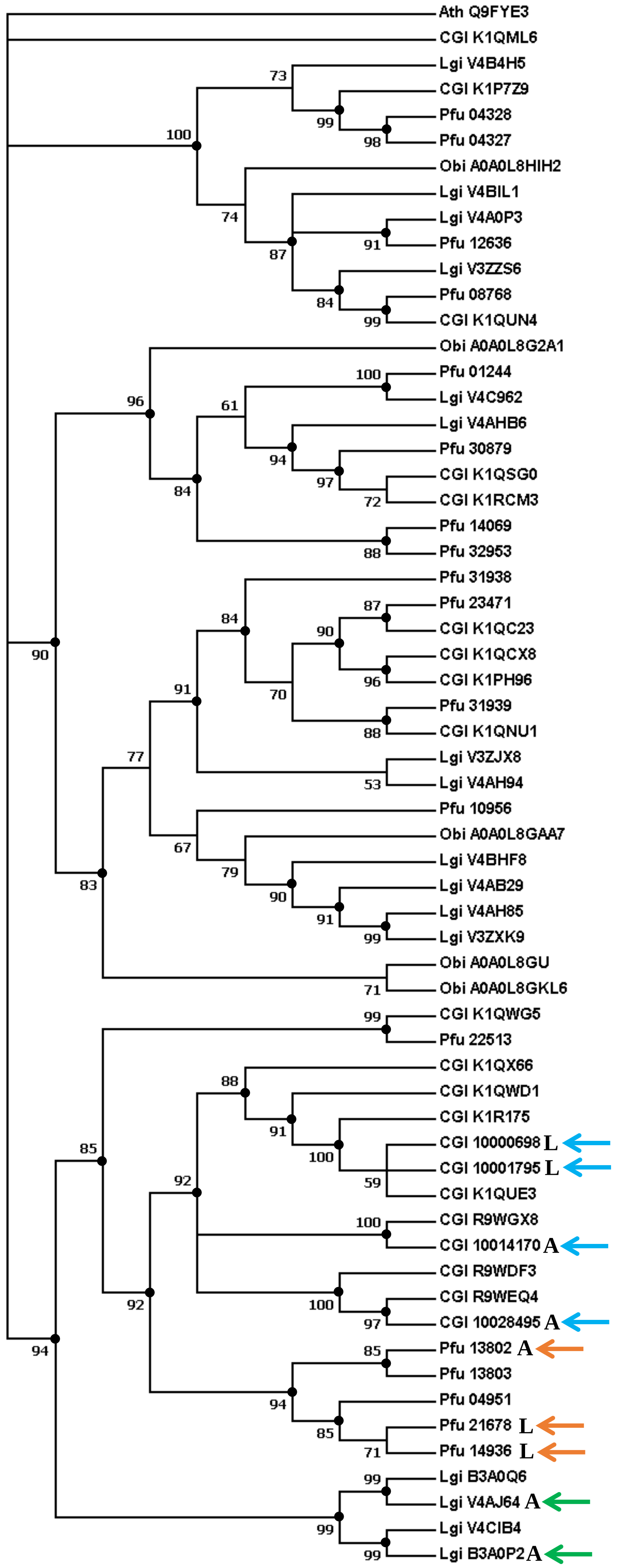

Ath Q9FYE3
CGI K1QML6
Lgi V4B4H5
CGI K1P7Z9
Pfu 04328
Pfu 04327
Obi A0A0L8HIH2
Lgi V4BIL1
Lgi V4A0P3
Pfu 12636
Lgi V3ZZS6
Pfu 08768
CGI K1QUN4
Obi A0A0L8G2A1
Pfu 01244
Lgi V4C962
Lgi V4AHB6
Pfu 30879
CGI K1QSG0
CGI K1RCM3
Pfu 14069
Pfu 32953
Pfu 31938
Pfu 23471
CGI K1QC23
CGI K1QCX8
CGI K1PH96
Pfu 31939
CGI K1QNU1
Lgi V3ZJX8
Lgi V4AH94
Pfu 10956
Obi A0A0L8GAA7
Lgi V4BHF8
Lgi V4AB29
Lgi V4AH85
Lgi V3ZXK9
Obi A0A0L8GU
Obi A0A0L8GKL6
CGI K1QWG5
Pfu 22513
CGI K1QX66
CGI K1QWD1
CGI K1R175
CGI 10000698 L
CGI 10001795 L
CGI K1QUE3
CGI R9WGX8
CGI 10014170 A
CGI R9WDF3
CGI R9WEQ4
CGI 10028495 A
Pfu 13802 A
Pfu 13803
Pfu 04951
Pfu 21678 L
Pfu 14936 L
Lgi B3A0Q6
Lgi V4AJ64 A
Lgi V4CIB4
Lgi B3A0P2 A

d

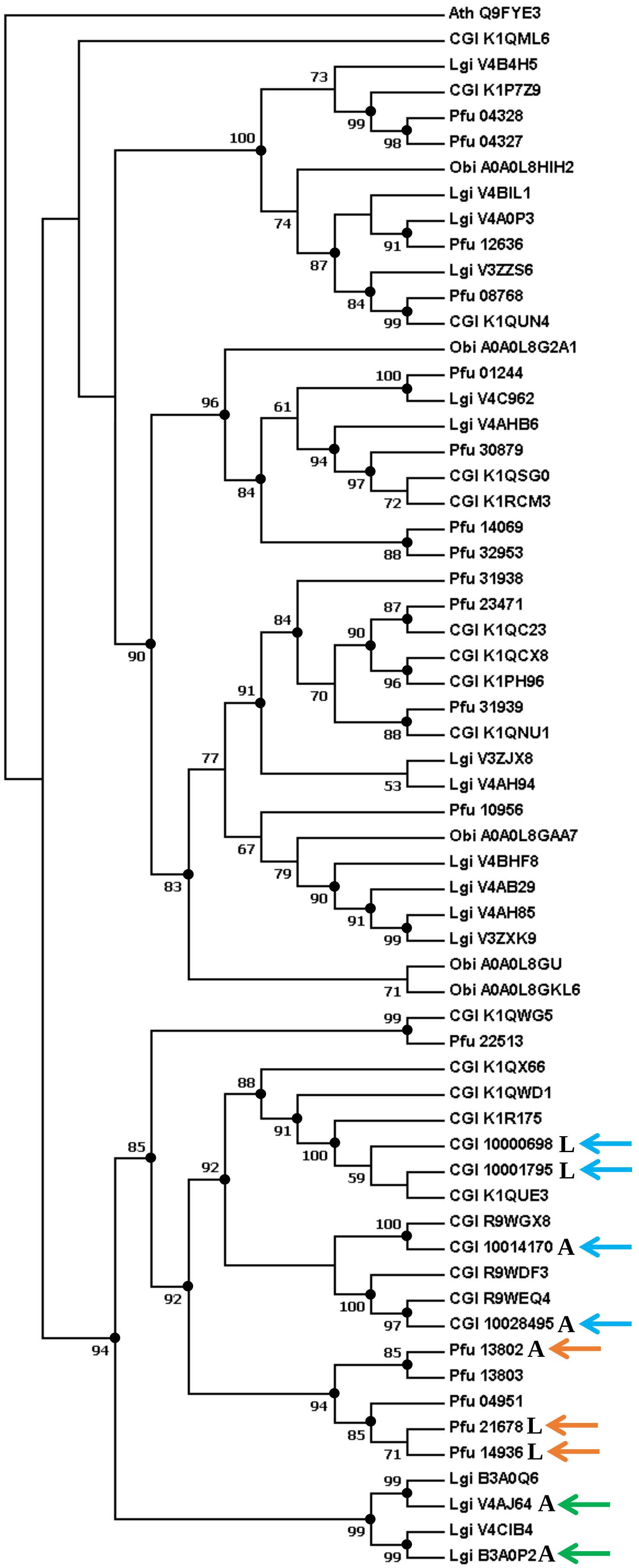

Supplementary Fig. 7 Maximum-likelihood trees generated via MEGA X (**a**, **b**) and PhyML (**c**, **d**) on 935 amino acid residues of CA domains in molluscs. SMPs are indicated by blue (*C. gigas*), orange (*P. fucata*) and green (*L. gigantea*) arrowheads. Larval and adult SMPs are marked by "L" and "A", respectively. Polychotomy is generated if the bootstrap value of the node is <50. Bootstrap values are shown if ≥50, and marked with black dots if ≥80. Cgi, *Crassostrea gigas*; Pfu, *Pinctada fucata*; Lgi, *Lottia gigantea*; Obi, *Octopus bimaculoides*. Ath, *Arabidopsis thaliana*.

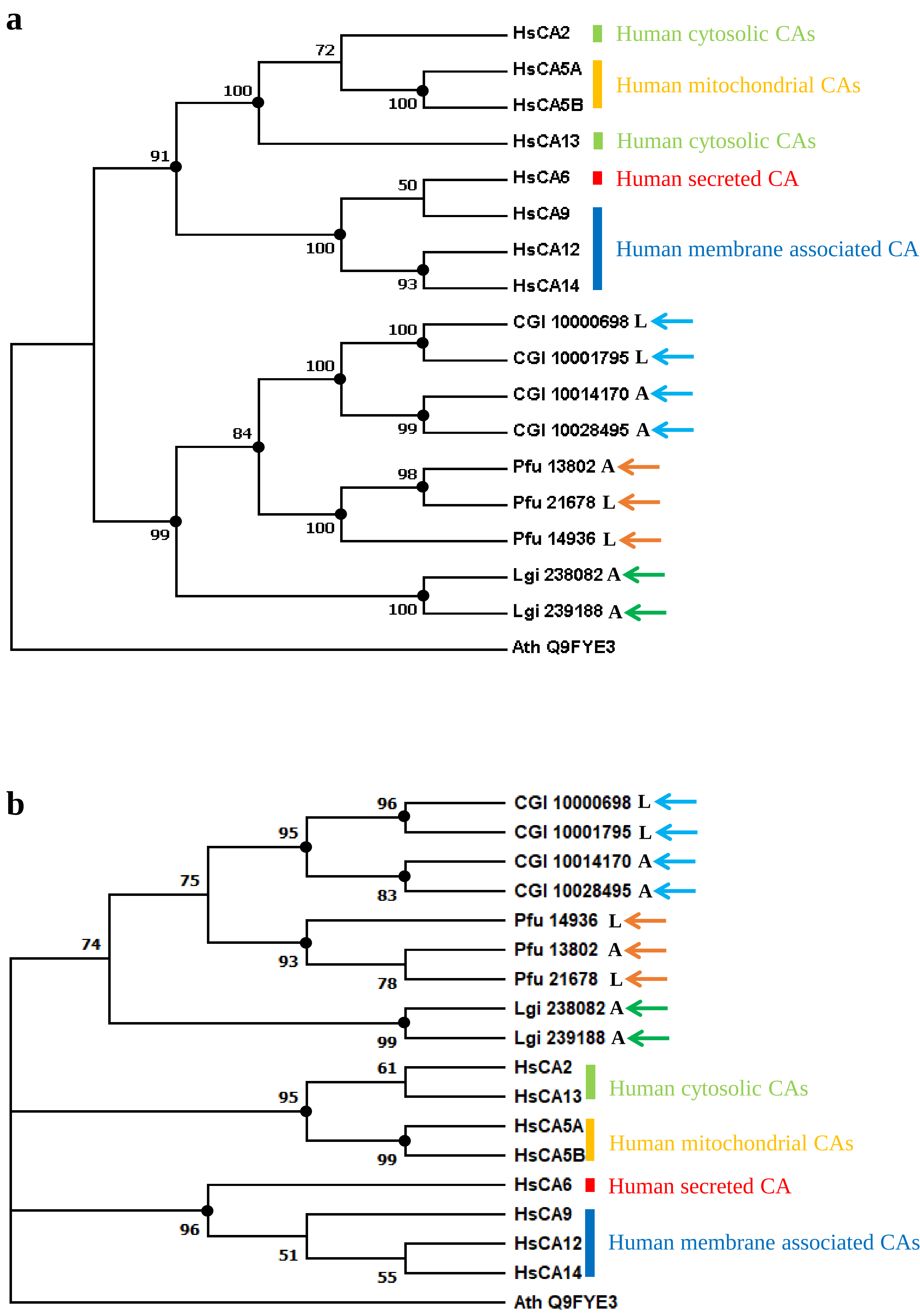
a
HsCA2
HsCA5A
HsCA5B
HsCA13
HsCA6
HsCA9
HsCA12
HsCA14
CGI 10000698 L
CGI 10001795 L
CGI 10014170 A
CGI 10028495 A
Pfu 13802 A
Pfu 21678 L
Pfu 14936 L
Lgi 238082 A
Lgi 239188 A
Ath Q9FYE3
Human cytosolic CAs
Human mitochondrial CAs
Human cytosolic CAs
Human secreted CA
Human membrane associated CA
72
100
100
91
50
100
93
100
100
99
84
98
100
99
100
b
CGI 10000698 L
CGI 10001795 L
CGI 10014170 A
CGI 10028495 A
Pfu 14936 L
Pfu 13802 A
Pfu 21678 L
Lgi 238082 A
Lgi 239188 A
HsCA2
HsCA13
HsCA5A
HsCA5B
HsCA6
HsCA9
HsCA12
HsCA14
Ath Q9FYE3
Human cytosolic CAs
Human mitochondrial CAs
Human secreted CA
Human membrane associated CAs
96
95
83
75
74
93
78
99
61
95
99
96
51
55

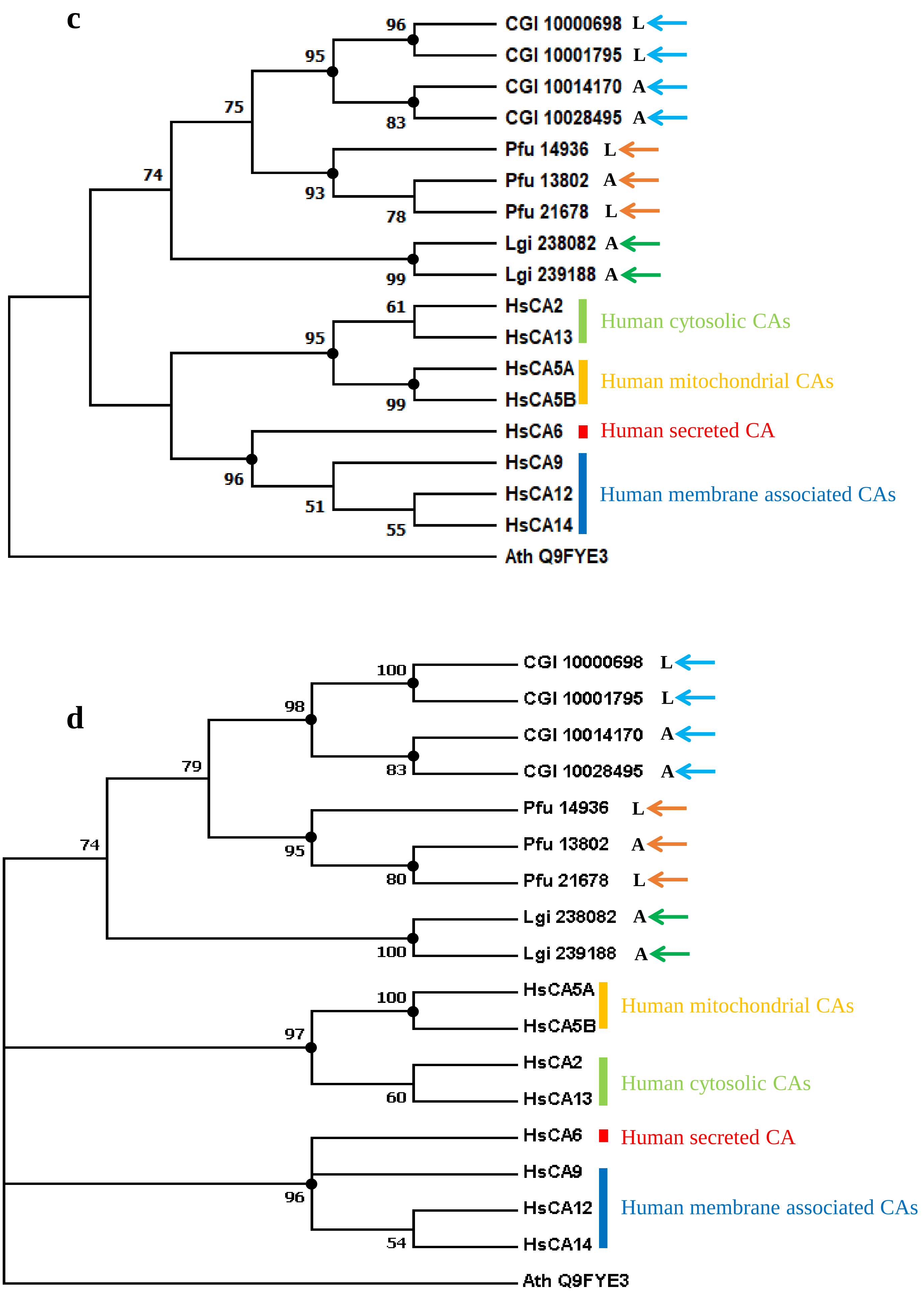

c
CGI 10000698 L
CGI 10001795 L
CGI 10014170 A
CGI 10028495 A
Pfu 14936 L
Pfu 13802 A
Pfu 21678 L
Lgi 238082 A
Lgi 239188 A
HsCA2
HsCA13
HsCA5A
HsCA5B
HsCA6
HsCA9
HsCA12
HsCA14
Ath Q9FYE3
Human cytosolic CAs
Human mitochondrial CAs
Human secreted CA
Human membrane associated CAs
96
95
75
83
74
93
78
99
61
95
99
96
51
55
d
CGI 10000698 L
CGI 10001795 L
CGI 10014170 A
CGI 10028495 A
Pfu 14936 L
Pfu 13802 A
Pfu 21678 L
Lgi 238082 A
Lgi 239188 A
HsCA5A
HsCA5B
HsCA2
HsCA13
HsCA6
HsCA9
HsCA12
HsCA14
Ath Q9FYE3
Human mitochondrial CAs
Human cytosolic CAs
Human secreted CA
Human membrane associated CAs
100
98
79
83
74
95
80
100
100
97
60
96
54

e

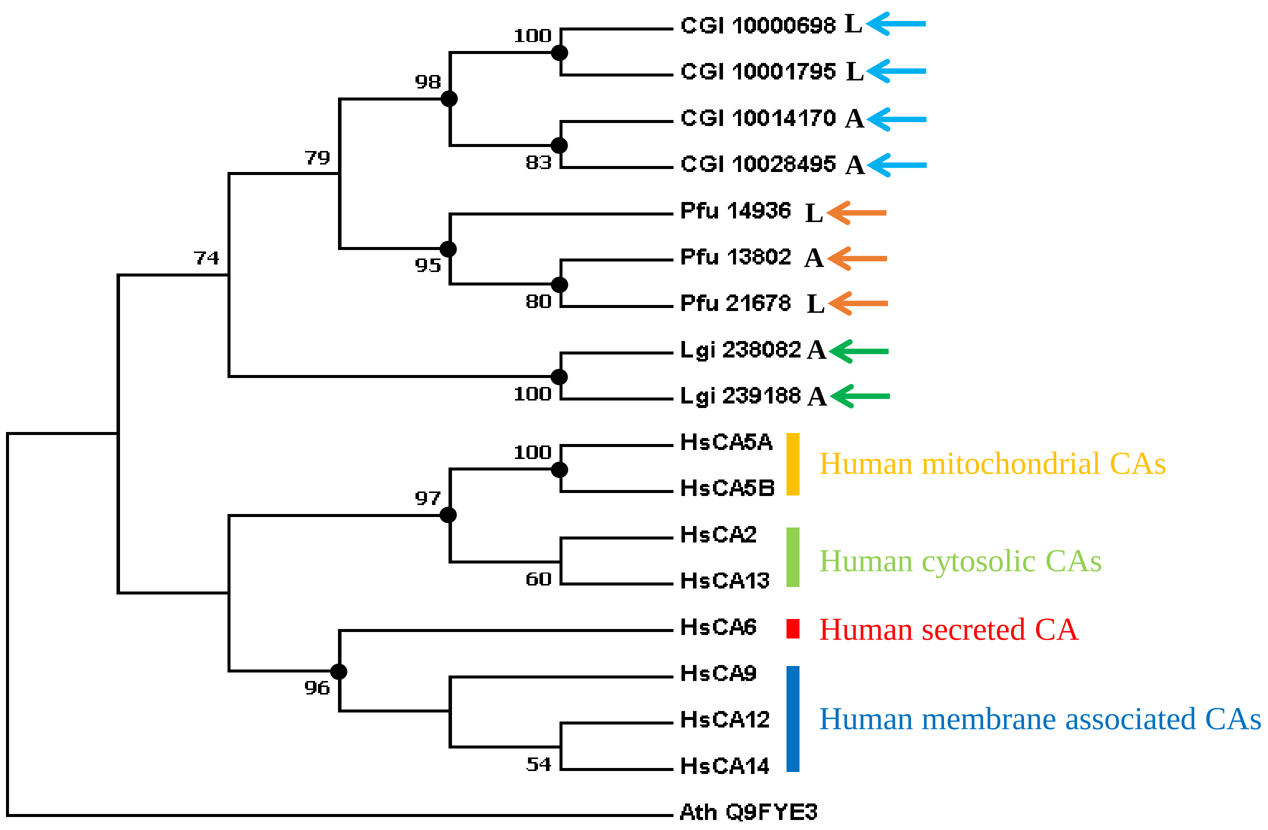

Supplementary Fig. 8 Phylogenetic analyses of CAs in molluscan shells with human CAs on 494 amino acid residues performed by MrBayes (**a**), MEGA X (**b, c**) and PhyML (**d**, **e**), respectively. **a** Polychotomy is generated if the posterior probability value of the node is <50. **b** Polychotomy is generated if the bootstrap value of the node is <50. **c** Original tree retaining dichotomies. **d** Polychotomy is generated if the bootstrap value of the node is <50. **e** Original tree retaining dichotomies. Posterior probability/Bootstrap values are shown if ≥50, and marked with black dots if ≥80. SMPs are indicated by blue (*C. gigas*), orange (*P. fucata*) and green (*L. gigantea*) arrowheads. Larval and adult SMPs are marked by “L” and “A”, respectively. CGI, *Crassostrea gigas*; Pfu, *Pinctada fucata*; Lgi, *Lottia gigantea*; Ath, *Arabidopsis thaliana*.

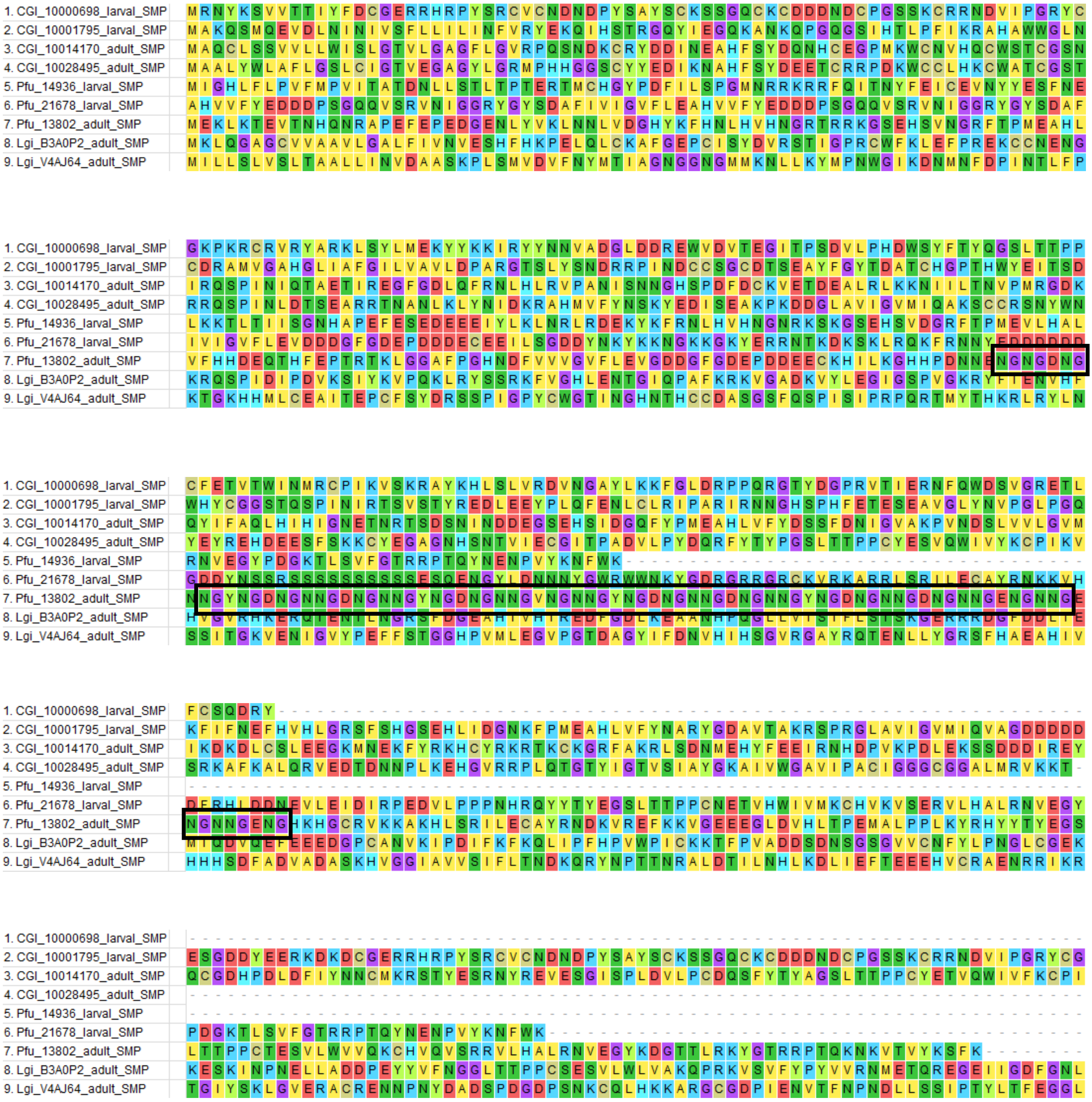
1. CGI_10000698_larval_SMP
2. CGI_10001795_larval_SMP
3. CGI_10014170_adult_SMP
4. CGI_10028495_adult_SMP
5. Pfu_14936_larval_SMP
6. Pfu_21678_larval_SMP
7. Pfu_13802_adult_SMP
8. Lgi_B3A0P2_adult_SMP
9. Lgi_V4AJ64_adult_SMP

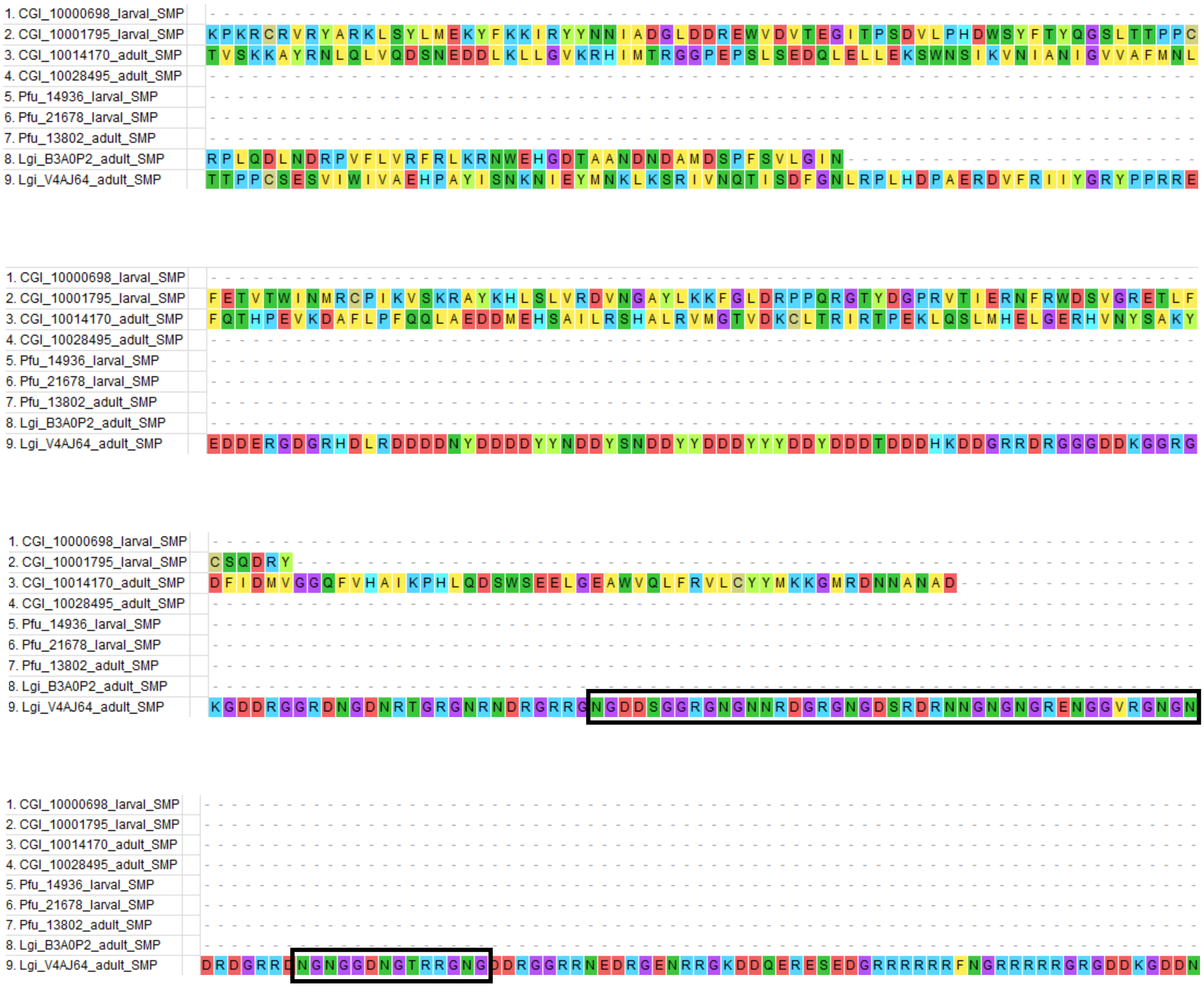

Supplementary Fig. 9 Alignment of molluscan CAs identified from the shells. NG-repeat domains are indicated by black boxes. Cgi, *Crassostrea gigas*; Pfu, *Pinctada fucata*; Lgi, *Lottia gigantea*. The alignment is viewed by MEGA X.
