# Supplementary material for: Phylogenetic comparisons reveal mosaic histories of larval and adult shell matrix protein deployment in pteriomorph bivalves
Source: Sci Rep. 2020 Dec 17;10:22140. doi: 10.1038/s41598-020-79330-x (PMC7747718; doi:10.1038/s41598-020-79330-x)
Supplement: Supplementary file 2 — Supplementary Tables. [file 41598_2020_79330_MOESM2_ESM.docx]

**Article** (Discoveries)

Phylogenetic comparisons reveal mosaic histories of larval and adult shell matrix protein deployment in pteriomorph bivalves
 
Ran Zhao^1,8,*^, Takeshi Takeuchi^2^, Ryo Koyanagi^3^, Alejandro Villar-Briones^4^, Lixy Yamada^5^, Hitoshi Sawada^5^, Akito Ishikawa^1^, Shunsuke Iwanaga^6^, Kiyohito Nagai^7^, Yuqi Che^8^, Noriyuki Satoh^2^ and Kazuyoshi Endo^1

1^ Department of Earth and Planetary Science, Graduate School of Science, University of Tokyo, Bunkyo-ku, Tokyo 113-0033, Japan
^2^ Marine Genomics Unit, Okinawa Institute of Science and Technology Graduate University, Onna, Okinawa, 904-0495 Japan
^3^ DNA Sequencing Section, Okinawa Institute of Science and Technology Graduate University, Onna, Okinawa, 904-0495 Japan
^4^ Instrumental Analysis Section, Okinawa Institute of Science and Technology Graduate University, Onna, Okinawa, 904-0495 Japan
^5^ Sugashima Marine Biological Laboratory, Graduate School of Science,
Nagoya University, Sugashima, Toba 517-0004, Japan
^6^ Nagasaki Prefectural Institute of Fisheries, Nagasaki, Nagasaki 851-2213, Japan
^7^ Pearl Research Institute, Mikimoto CO., LTD, Shima, Mie 517-0403, Japan

^8^Department of Biology, Shenzhen MSU-BIT University, 1 International University Park Road, Dayun New Town, Longgang District, Shenzhen, Guangdong Province, P.R. China
^*^Correspondence: [zhao_ran13@hotmail.com](mailto:zhao_ran13@hotmail.com) (Ran. Zhao)

**Supplementary Table 1** Number of genes encoding functional domains in animal genomes.

| Phylum | Species | VWA(IPR002035) | Chitin binding domain （IPR002557） | VWA(IPR002035)+ Chitin binding  domain （IPR002557） |
| --- | --- | --- | --- | --- |
| Mollusca | *Pinctada fucata* | 193 | 85 | 19 |
|  | *Crassostrea gigas* | 158 | 86 | 16 |
|  | *Bathymodiolus platifrons* | 160 | 79 | 7 |
|  | *Modiolus philippinarum* | 183 | 79 | 8 |
|  | *Patinopecten yessoensis* | 234 | 124 | 17 |
|  | *Chlamys farreri* | 144 | 88 | 14 |
|  | *Lottia gigantea* | 91 | 93 | 6 |
|  | *Haliotis discus hannai* | 89 | 95 | 3 |
|  | *Aplysia californica* | 136 | 108 | 14 |
|  | *Biomphalaria glabrata* | 154 | 94 | 8 |
|  | *Octopus bimaculoides* | 118 | 78 | 2 |
| Nemertea | *Notospermus geniculatus* | 165 | 17 | 2 |
| Phoronida | *Phoronis australis* | 106 | 38 | 2 |
| Brachiopoda | *Lingula anatina* | 192 | 154 | 6 |
| Annelida | *Capitella teleta* | 102 | 85 | 0 |
|  | *Helobdella rubusta* | 44 | 14 | 0 |
| Platyhelminthes | *Schmidtea mediterranea* | 33 | 0 | 0 |
|  | *Schistosoma mansoni* | 10 | 1 | 0 |
| Rotifera | *Adineta vaga* | 113 | 47 | 0 |
| Nematoda | *Caenorhabditis elegans* | 83 | 16 | 0 |
| Arthropoda | *Drosophila melanogaster* | 29 | 167 | 0 |
|  | *Tribolium castaneum* | 21 | 76 | 0 |
| Echinodermata | *Strongylocentrotus purpuratus* | 69 | 18 | 0 |
| Chordata | *Ciona intestinalis* | 102 | 12 | 1 |
|  | *Branchiostoma floridae* | 284 | 59 | 2 |
|  | *Danio rerio* | 209 | 8 | 0 |
|  | *Gallus gallus* | 96 | 6 | 0 |
|  | *Mus musculus* | 236 | 8 | 0 |
|  | *Homo sapiens* | 356 | 13 | 0 |
| Cnidaria | *Acropora digtifera* | 117 | 11 | 0 |
|  | *Aiptasia* | 156 | 24 | 0 |
|  | *Nematostella vectensis* | 119 | 21 | 0 |
|  | *Hydra magnipapillata* | 67 | 43 | 0 |
| Porifera | *Amphimedon queenslandica* | 55 | 2 | 0 |
| Placozoa | *Tricholax adherens* | 50 | 2 | 0 |
| Choanzoa | *Monosiga brevicollis* | 22 | 0 | 0 |

This is a restated table. Please refer to our previous research^1^ for more detailed information.

**Supplementary Table 2** List of VWA-CB dcps subjected to phylogenetic analyses in this study.

| ID/Protein | Larval SMP | Adult SMP | Species | Accession code | Reference  /Resource |
| --- | --- | --- | --- | --- | --- |
| CGI_10017473/VWA-CB dcp | Yes |  | *Crassostrea gigas* | CGI_10017473 | [^1^](#_ENREF_1) |
| CGI_10028014/VWA-CB dcp |  | Yes | *Crassostrea gigas* | CGI_10028014 | [^2^](#_ENREF_2) |
| CGI_10009194/BMSP | Yes |  | *Crassostrea gigas* | CGI_10009194 | [^1^](#_ENREF_1) |
| Pfu_21296/VWA-CB dcp | Yes |  | *Pinctada fucata* | Pfu_aug2.0_956.1_21296.t1 | [^1^](#_ENREF_1) |
| Pfu_04155/VWA-CB dcp | Yes |  | *Pinctada fucata* | Pfu_aug2.0_421.1_04155.t1 | [^1^](#_ENREF_1) |
| Pfu_09248/VWA-CB dcp |  | Yes | *Pinctada fucata* | Pfu_aug2.0_3932.1_09248.t1 | [^1^](#_ENREF_1) |
| Pfu-Pif/Pif |  | Yes | *Pinctada fucata* | Pfu_aug2.0_715.1_17768.t1 | [^1^](#_ENREF_1) |
| Pfu_30448/VWA-CB dcp |  | Yes | *Pinctada fucata* | Pfu_aug2.0_219.1_30448.t1 | [^1^](#_ENREF_1) |
| Pfu-BMSP/BMSP | Yes | Yes | *Pinctada fucata* | Pfu_cdna2.0_089203 | [^1^](#_ENREF_1) |
| Mga-BMSP/BMSP |  | Yes | *Mytilus galloprovincialis* | BAK86420.1 | [^3^](#_ENREF_3) |
| Lgi_228264/VWA-CB dcp |  | Yes | *Lottia gigantea* | Lgi_228264 | [^4^](#_ENREF_4) |
| Lgi_232022/VWA-CB dcp |  | Yes | *Lottia gigantea* | Lgi_232022 | [^4^](#_ENREF_4) |
| Lgi-BMSP/BMSP |  | Yes | *Lottia gigantea* | Lgi_173137, 173138, 173139 | [^5^](#_ENREF_5) |
| Lan1530/VWA-CB dcp |  |  | *Lingular anatina* | XP_013393007.1 | 8 |

**Supplementary Table 3** List of chitobiase-like proteins analyzed phylogenetically in this study.

| Protein ID/Protein | Larval SMP | Adult SMP | Species | Accession code | Reference |
| --- | --- | --- | --- | --- | --- |
| CGI_10007856/[*N*,*N*'-diacetylchitobiase](https://blast.ncbi.nlm.nih.gov/Blast.cgi#alnHdr_405951819) | Yes |  | *Crassostrea gigas* | CGI_10007856 | [^1^](#_ENREF_1) |
| CGI_10007857/[Chitobiase](https://blast.ncbi.nlm.nih.gov/Blast.cgi#alnHdr_405951820) |  | Yes | *Crassostrea gigas* | CGI_10007857 | [^2^](#_ENREF_2) |
| CGI_10002999/*N*,*N*'-diacetylchitobiase |  |  | *Crassostrea gigas* | CGI_10002999 | [^2^](#_ENREF_2) |
| CGI_10023816/[Chitobiase](https://blast.ncbi.nlm.nih.gov/Blast.cgi#alnHdr_405963894) |  |  | *Crassostrea gigas* | CGI_10023816 | [^2^](#_ENREF_2) |
| CGI_10027871/[*N*,*N*'-diacetylchitobiase](https://blast.ncbi.nlm.nih.gov/Blast.cgi#alnHdr_405975668) |  |  | *Crassostrea gigas* | CGI_10027871 | [^2^](#_ENREF_2) |
| Pfu_20027/[*N*,*N*'-diacetylchitobiase](https://blast.ncbi.nlm.nih.gov/Blast.cgi#alnHdr_405951819) | Yes |  | *Pinctada fucata* | pfu_aug2.0_6.1_20027.t1 | [^1^](#_ENREF_1) |
| Pfu_20028/[Beta-hexosaminidase](https://blast.ncbi.nlm.nih.gov/Blast.cgi#alnHdr_1207913632) |  | Yes | *Pinctada fucata* | pfu_aug2.0_6.1_20028.t1 | [^1^](#_ENREF_1) |
| Pfu_08823/[Chitobiase](https://blast.ncbi.nlm.nih.gov/Blast.cgi#alnHdr_405951820) |  |  | *Pinctada fucata* | pfu_aug2.0_2312.1_08823.t1 | [^6^](#_ENREF_6) |
| Pfu_15958/[Chitobiase](https://blast.ncbi.nlm.nih.gov/Blast.cgi#alnHdr_405951820) |  |  | *Pinctada fucata* | pfu_aug2.0_4334.1_15958.t1 | [^6^](#_ENREF_6) |
| Pfu_31002/[Chitobiase](https://blast.ncbi.nlm.nih.gov/Blast.cgi#alnHdr_405951820) |  |  | *Pinctada fucata* | pfu_aug2.0_629.1_31002.t1 | [^6^](#_ENREF_6) |
| Lgi_151739/[Chitobiase](https://blast.ncbi.nlm.nih.gov/Blast.cgi#alnHdr_405951820) |  |  | *Lottia gigantea* | Lgi_151739 | [^7^](#_ENREF_7) |
| Lgi_168843/[Chitobiase](https://blast.ncbi.nlm.nih.gov/Blast.cgi#alnHdr_405951820) |  |  | *Lottia gigantea* | Lgi_168843 | [^7^](#_ENREF_7) |
| Lgi_174972/[Chitobiase](https://blast.ncbi.nlm.nih.gov/Blast.cgi#alnHdr_405951820) |  |  | *Lottia gigantea* | Lgi_174972 | [^7^](#_ENREF_7) |
| Lgi_236342/[Chitobiase](https://blast.ncbi.nlm.nih.gov/Blast.cgi#alnHdr_405951820) |  |  | *Lottia gigantea* | Lgi_236342 | [^7^](#_ENREF_7) |
| Lgi_239375/[Chitobiase](https://blast.ncbi.nlm.nih.gov/Blast.cgi#alnHdr_405951820) |  |  | *Lottia gigantea* | Lgi_239375 | [^7^](#_ENREF_7) |
| Obi_014767869/[Beta-hexosaminidase-like isoform X1](https://blast.ncbi.nlm.nih.gov/Blast.cgi#alnHdr_1207913632) |  |  | *Octopus bimaculoides* | XP_014767869.1 | ^17^ |
| Obi_014767876/[Beta-hexosaminidase-like isoform X2](https://blast.ncbi.nlm.nih.gov/Blast.cgi#alnHdr_1207913632) |  |  | *Octopus bimaculoides* | XP_014767876.1 | ^17^ |
| Obi_014767885/[Beta-hexosaminidase-like isoform X3](https://blast.ncbi.nlm.nih.gov/Blast.cgi#alnHdr_1207913632) |  |  | *Octopus bimaculoides* | XP_014767885.1 | ^17^ |
| Obi_014790058/[*N*,*N*'-diacetylchitobiase-like](https://blast.ncbi.nlm.nih.gov/Blast.cgi#alnHdr_405951819) |  |  | *Octopus bimaculoides* | XP_014790058.1 | ^17^ |
| Lan_013407630/[Chitobiase](https://blast.ncbi.nlm.nih.gov/Blast.cgi#alnHdr_405951820) |  |  | *Lingula anatina* | XP_013407630.1 | [^8^](#_ENREF_8) |

**Supplementary Table 4** List of proteins containing Glyco_18 domain (IPR011583) or

Glyco_hydro_20 domain (IPR015883) identified in the shell proteome data and genome data of *C.*

*gigas* and *P. fucata*.

|  | Glyco_18 domain (IPR011583) | Glyco_hydro_20 domain (IPR015883) | Reference |
| --- | --- | --- | --- |
| Larval shell proteome of *C. gigas* |  | CGI_10007856 ？ | [^1^](#_ENREF_1) |
| Adult shell proteome of *C. gigas* | CGI_10026605 | CGI_10007857 | [^2^](#_ENREF_2) |
| Larval shell proteome of *P. fucata* |  | pfu_aug2.0_6.1_20027.t1 | [^1^](#_ENREF_1) |
| Adult shell proteome of *P. fucata* | pfu_aug2.0_194.1_13762.t1 | pfu_aug2.0_6.1_20028.t1 | [^1^](#_ENREF_1) |
|  | pfu_aug2.0_194.1_13763.t1 |  | [^1^](#_ENREF_1) |
| Genome of *C. gigas* | CGI_10002421 | CGI_10001718 | [^2^](#_ENREF_2) |
|  | CGI_10006211 | CGI_10002246 | [^2^](#_ENREF_2) |
|  | CGI_10022102 | CGI_10002999 | [^2^](#_ENREF_2) |
|  | CGI_10022487 | CGI_10004764 | [^2^](#_ENREF_2) |
|  | CGI_10024867 | CGI_10007196 | [^2^](#_ENREF_2) |
|  | CGI_10024868 | CGI_10007856 | [^2^](#_ENREF_2) |
|  | CGI_10024869 | CGI_10007857 | [^2^](#_ENREF_2) |
|  | CGI_10024870 | CGI_10008618 | [^2^](#_ENREF_2) |
|  | CGI_10026598 | CGI_10017246 | [^2^](#_ENREF_2) |
|  | CGI_10026599 | CGI_10022781 | [^2^](#_ENREF_2) |
|  | CGI_10026600 | CGI_10023604 | [^2^](#_ENREF_2) |
|  | CGI_10026601 | CGI_10023605 | [^2^](#_ENREF_2) |
|  | CGI_10026602 | CGI_10023816 | [^2^](#_ENREF_2) |
|  | CGI_10026603 | CGI_10024502 | [^2^](#_ENREF_2) |
|  | CGI_10026604 | CGI_10027871 | [^2^](#_ENREF_2) |
|  | CGI_10026605 | CGI_10028163 | [^2^](#_ENREF_2) |
|  | CGI_10026762 |  | [^2^](#_ENREF_2) |
| Genome of *P. fucata* | pfu_aug2.0_11054.1_16443.t1 | pfu_aug2.0_2312.1_08823.t1 | [^6^](#_ENREF_6) |
|  | pfu_aug2.0_1382.1_08349.t1 | pfu_aug2.0_25.1_16729.t1 | [^6^](#_ENREF_6) |
|  | pfu_aug2.0_14217.1_26475.t1 | pfu_aug2.0_2583.1_12215.t1 | [^6^](#_ENREF_6) |
|  | pfu_aug2.0_1660.1_01750.t1 | pfu_aug2.0_32.1_06783.t1 | [^6^](#_ENREF_6) |
|  | pfu_aug2.0_1664.1_15180.t1 | pfu_aug2.0_4334.1_15958.t1 | [^6^](#_ENREF_6) |
|  | pfu_aug2.0_1921.1_05318.t1 | pfu_aug2.0_6.1_20027.t1 | [^6^](#_ENREF_6) |
|  | pfu_aug2.0_194.1_13761.t1 | pfu_aug2.0_6.1_20028.t1 | [^6^](#_ENREF_6) |
|  | pfu_aug2.0_194.1_13762.t1 | pfu_aug2.0_629.1_31002.t1 | [^6^](#_ENREF_6) |
|  | pfu_aug2.0_194.1_13763.t1 | pfu_aug2.0_63.1_10205.t2 | [^6^](#_ENREF_6) |
|  | pfu_aug2.0_194.1_13766.t1 |  | [^6^](#_ENREF_6) |
|  | pfu_aug2.0_21.1_03434.t1 |  | [^6^](#_ENREF_6) |
|  | pfu_aug2.0_21.1_03435.t1 |  | [^6^](#_ENREF_6) |
|  | pfu_aug2.0_2404.1_15511.t1 |  | [^6^](#_ENREF_6) |
|  | pfu_aug2.0_2934.1_15667.t1 |  | [^6^](#_ENREF_6) |
|  | pfu_aug2.0_3570.1_02442.t1 |  | [^6^](#_ENREF_6) |
|  | pfu_aug2.0_620.1_00959.t1 |  | [^6^](#_ENREF_6) |
|  | pfu_aug2.0_70.1_00174.t1 |  | [^6^](#_ENREF_6) |
|  | pfu_aug2.0_84.1_13566.t1 |  | [^6^](#_ENREF_6) |
|  | pfu_aug2.0_84.1_13567.t1 |  | [^6^](#_ENREF_6) |
|  | pfu_aug2.0_903.1_11297.t1 |  | [^6^](#_ENREF_6) |

The question mark indicates whether or not the gene is encoding an SMP is uncertain.

**Supplementary Table 5** List of proteins containing carbonic anhydrase domain applied to phylogenetic analyses in this study.

| Protein ID/Protein | Larval SMP | Adult SMP | Species | Accession code | Reference/Resource |
| --- | --- | --- | --- | --- | --- |
| CGI_10000698/Carbonic anhydrase | Yes |  | *Crassostrea gigas* | K1Q802/CGI_10000698 | [^2^](#_ENREF_2) |
| CGI_10001795/Carbonic anhydrase | Yes |  | *Crassostrea gigas* | K1QM00/CGI_10001795 | [^2^](#_ENREF_2) |
| CGI_10014170/Carbonic anhydrase |  | Yes | *Crassostrea gigas* | K1RJ02/CGI_10014170 | [^2^](#_ENREF_2) |
| Cgi_10028495/Carbonic anhydrase |  | Yes | *Crassostrea gigas* | K1S2Z6/CGI_10028495 | [^2^](#_ENREF_2) |
| CGI_ K1P7Z9/CA domain-containing protein |  |  | *Crassostrea gigas* | K1P7Z9 | [^2^](#_ENREF_2) |
| CGI_ K1PH96/Carbonic anhydrase |  |  | *Crassostrea gigas* | K1PH96 | [^2^](#_ENREF_2) |
| CGI_ K1QC23/Carbonic anhydrase 1 |  |  | *Crassostrea gigas* | K1QC23 | [^2^](#_ENREF_2) |
| CGI_ K1QCX8/Carbonic anhydrase 12 |  |  | *Crassostrea gigas* | K1QCX8 | [^2^](#_ENREF_2) |
| CGI_ K1QNU1/Carbonic anhydrase 2 |  |  | *Crassostrea gigas* | K1QNU1 | [^2^](#_ENREF_2) |
| CGI_ K1QSG0/Carbonic anhydrase 2 |  |  | *Crassostrea gigas* | K1QSG0 | [^2^](#_ENREF_2) |
| CGI_ K1QUE3/Nacrein-like protein P2 |  |  | *Crassostrea gigas* | K1QUE3 | [^2^](#_ENREF_2) |
| CGI_ K1QUN4/DnaJ-like protein subfamily C member 7 |  |  | *Crassostrea gigas* | K1QUN4 | [^2^](#_ENREF_2) |
| CGI_ K1QWD1/Carbonic anhydrase 2 |  |  | *Crassostrea gigas* | K1QWD1 | [^2^](#_ENREF_2) |
| CGI_ K1QX66/Carbonic anhydrase 2 |  |  | *Crassostrea gigas* | K1QX66 | [^2^](#_ENREF_2) |
| CGI_ K1R175/Carbonic anhydrase 7 |  |  | *Crassostrea gigas* | K1R175 | [^2^](#_ENREF_2) |
| CGI_ K1RCM3/Carbonic anhydrase 2 |  |  | *Crassostrea gigas* | K1RCM3 | [^2^](#_ENREF_2) |
| CGI_ K1RKE6/Carbonic anhydrase 13 |  |  | *Crassostrea gigas* | K1RKE6 | [^2^](#_ENREF_2) |
| CGI_ R9WDF3/Nacrein-like protein F2 |  |  | *Crassostrea gigas* | R9WDF3 | [^9^](#_ENREF_9) |
| CGI_ R9WEQ4/Nacrein-like protein |  |  | *Crassostrea gigas* | R9WEQ4 | ^18^ |
| CGI_ R9WGX8/Nacrein-like protein F1 |  |  | *Crassostrea gigas* | R9WGX8 | [^9^](#_ENREF_9) |
| Pfu_ 01244/CA domain-containing protein |  |  | *Pinctada fucata* | Pfu_aug2.0_920.1_01244.t2 | [^6^](#_ENREF_6) |
| Pfu_ 04951/CA domain-containing protein |  |  | *Pinctada fucata* | Pfu_aug2.0_1301.1_04951.t1 | [^6^](#_ENREF_6) |
| Pfu_ 04327/CA domain-containing protein |  |  | *Pinctada fucata* | Pfu_aug2.0_551.1_04327.t1 | [^6^](#_ENREF_6) |
| Pfu_ 04328/CA domain-containing protein |  |  | *Pinctada fucata* | Pfu_aug2.0_551.1_04328.t1 | [^6^](#_ENREF_6) |
| Pfu_ 08768/CA domain-containing protein |  |  | *Pinctada fucata* | Pfu_aug2.0_2182.1_08768.t1 | [^6^](#_ENREF_6) |
| Pfu_ 10956/CA domain-containing protein |  |  | *Pinctada fucata* | Pfu_aug2.0_583.1_10956.t1 | [^6^](#_ENREF_6) |
| Pfu_ 12636/CA domain-containing protein |  |  | *Pinctada fucata* | Pfu_aug2.0_4343.1_12636.t1 | [^6^](#_ENREF_6) |
| Pfu_ 13802/CA domain-containing protein |  | Yes | *Pinctada fucata* | Pfu_aug2.0_214.1_13802.t1 | [^1^](#_ENREF_1) |
| Pfu_ 13803/CA domain-containing protein |  |  | *Pinctada fucata* | Pfu_aug2.0_214.1_13803.t1 | [^6^](#_ENREF_6) |
| Pfu_ 14069/CA domain-containing protein |  |  | *Pinctada fucata* | Pfu_aug2.0_374.1_14069.t1 | [^6^](#_ENREF_6) |
| Pfu_ 14936/Carbonic anhydrase | YES |  | *Pinctada fucata* | Pfu_aug2.0_1294.1_14936.t1 | [^1^](#_ENREF_1) |
| Pfu_ 21678/Carbonic anhydrase | YES |  | *Pinctada fucata* | Pfu_aug2.0_1536.1_21678.t1 | [^1^](#_ENREF_1) |
| Pfu_ 22513/CA domain-containing protein |  |  | *Pinctada fucata* | Pfu_aug2.0_4146.1_22513.t1 | [^6^](#_ENREF_6) |
| Pfu_ 23471/CA domain-containing protein |  |  | *Pinctada fucata* | Pfu_aug2.0_107.1_23471.t1 | [^6^](#_ENREF_6) |
| Pfu_ 25196/CA domain-containing protein |  |  | *Pinctada fucata* | Pfu_aug2.0_1907.1_25196.t1 | [^6^](#_ENREF_6) |
| Pfu_ 30879/CA domain-containing protein |  |  | *Pinctada fucata* | Pfu_aug2.0_539.1_30879.t1 | [^6^](#_ENREF_6) |
| Pfu_ 31938/CA domain-containing protein |  |  | *Pinctada fucata* | Pfu_aug2.0_1849.1_31938.t1 | [^6^](#_ENREF_6) |
| Pfu_ 31939/CA domain-containing protein |  |  | *Pinctada fucata* | Pfu_aug2.0_1849.1_31939.t1 | [^6^](#_ENREF_6) |
| Pfu_ 32953/CA domain-containing protein |  |  | *Pinctada fucata* | Pfu_aug2.0_7459.1_32953.t1 | [^6^](#_ENREF_6) |
| Lgi_ B3A0P2/Putative carbonic anhydrase 1 |  | Yes | *Lottia gigantea* | B3A0P2 | [^5^](#_ENREF_5) |
| Lgi_ B3A0Q6/Putative carbonic anhydrase 2 |  |  | *Lottia gigantea* | B3A0Q6 | [^5^](#_ENREF_5) |
| Lgi_ V3ZJX8/CA domain-containing protein |  |  | *Lottia gigantea* | V3ZJX8 | [^7^](#_ENREF_7) |
| Lgi_ V3ZXK9/CA domain-containing protein |  |  | *Lottia gigantea* | V3ZXK9 | [^7^](#_ENREF_7) |
| Lgi_ V3ZZS6/CA domain-containing protein |  |  | *Lottia gigantea* | V3ZZS6 | [^7^](#_ENREF_7) |
| Lgi_ V4A0P3/CA domain-containing protein |  |  | *Lottia gigantea* | V4A0P3 | [^7^](#_ENREF_7) |
| Lgi_ V4AB29/CA domain-containing protein |  |  | *Lottia gigantea* | V4AB29 | [^7^](#_ENREF_7) |
| Lgi_ V4AH85/CA domain-containing protein |  |  | *Lottia gigantea* | V4AH85 | [^7^](#_ENREF_7) |
| Lgi_ V4AH94/CA domain-containing protein |  |  | *Lottia gigantea* | V4AH94 | [^7^](#_ENREF_7) |
| Lgi_ V4AHB6/CA domain-containing protein |  |  | *Lottia gigantea* | V4AHB6 | [^7^](#_ENREF_7) |
| Lgi_ V4AJ64/CA domain-containing protein |  | Yes | *Lottia gigantea* | V4AJ64 | [^7^](#_ENREF_7) |
| Lgi_ V4B4H5/CA domain-containing protein |  |  | *Lottia gigantea* | V4B4H5 | [^7^](#_ENREF_7) |
| Lgi_ V4BHF8/CA domain-containing protein |  |  | *Lottia gigantea* | V4BHF8 | [^7^](#_ENREF_7) |
| Lgi_ V4BIL1/CA domain-containing protein |  |  | *Lottia gigantea* | V4BIL1 | [^7^](#_ENREF_7) |
| Lgi_ V4C962/CA domain-containing protein |  |  | *Lottia gigantea* | V4C962 | [^7^](#_ENREF_7) |
| Lgi_ V4CIB4/CA domain-containing protein |  |  | *Lottia gigantea* | V4CIB4 | [^7^](#_ENREF_7) |
| Obi_ A0A0L8G2A1/CA domain-containing protein |  |  | *Octopus bimaculoides* | A0A0L8G2A1 | ^17^ |
| Obi_ A0A0L8GAA7/CA domain-containing protein |  |  | *Octopus bimaculoides* | A0A0L8GAA7 | ^17^ |
| Obi_ A0A0L8GKL6/CA domain-containing protein |  |  | *Octopus bimaculoides* | A0A0L8GKL6 | ^17^ |
| Obi_ A0A0L8GUI8/CA domain-containing protein |  |  | *Octopus bimaculoides* | A0A0L8GUI8 | ^17^ |
| Obi_ A0A0L8HIH2/CA domain-containing protein |  |  | *Octopus bimaculoides* | A0A0L8HIH2 | ^17^ |
| Ath_ Q9FYE3/Alpha carbonate dehydratase 3 |  |  | *Arabidopsis thaliana* | Q9FYE3 | [^10^](#_ENREF_10) |
| Hs_CA2/Cytosolic carbonic anhydrase |  |  | *Homo sapiens* | P00918 | [^11^](#_ENREF_11) |
| Hs_CA5A/Mitochondrial carbonic anhydrase |  |  | *Homo sapiens* | P35218 | [^12^](#_ENREF_12) |
| Hs_CA5B/Mitochondrial carbonic anhydrase |  |  | *Homo sapiens* | Q9Y2D0 | [^13^](#_ENREF_13) |
| Hs_CA6/Secreted carbonic anhydrase |  |  | *Homo sapiens* | P23280 | [^14^](#_ENREF_14) |
| Hs_CA9/Membrane associated carbonic anhydrase |  |  | *Homo sapiens* | Q16790 | ^15^ |
| Hs_CA12/Membrane associated carbonic anhydrase |  |  | *Homo sapiens* | O43570 | ^16^ |
| Hs_CA13/Cytosolic carbonic anhydrase |  |  | *Homo sapiens* | Q8N1Q1 | ^16^ |
| Hs_CA14/Membrane associated carbonic anhydrase |  |  | *Homo sapiens* | Q9ULX7 | ^16^ |

**Supplementary Table 6** Models applied to different maximum-likelihood phylogenetic analysis methods on each dataset.

| Alignment | MEGA X  (predicted by MEGA X) | PhyML  (predicted by ProtTest 3.4.2) | | |
| --- | --- | --- | --- | --- |
|  | Model | Model | Proportion of invariant sites | Gamma distribution parameter |
| CB | WAG+I+G | WAG+I+G | 5.071 | 0.076 |
| VWA | LG+G | LG+I+G | 1.78 | 0.006 |
| VWA(with Lgi236719) | LG+G | LG+G | 1.723 | 0.007 |
| Lamnin G | LG+G | LG+G | 2.864 | 0.012 |
| CB1&Lamnin G | WAG+G | W+I+G | 3.691 | 0.021 |
| CHB_HEX& Glyco_hydro_20b& Glyco_hydro_20& CHB_HEX_C | LG+G | LG+I+G | 1.409 | 0.023 |
| CA | WAG+G+F | WAG+I+G+F | 1.426 | 0.005 |
| CA(with human CA) | WAG+G+F | WAG+I+G+F | 2.041 | 0.002 |

**References**

1 Zhao, R. *et al.* Dual gene repertoires for larval and adult shells reveal molecules essential for molluscan shell formation. *Molecular biology and evolution* **35**, 2751-2761 (2018).

2 Zhang, G. *et al.* The oyster genome reveals stress adaptation and complexity of shell formation. *Nature* **490**, 49, doi:10.1038/nature11413

https://[www.nature.com/articles/nature11413#supplementary-information](http://www.nature.com/articles/nature11413#supplementary-information) (2012).

3 Suzuki, M. *et al.* Identification and Characterisation of a Calcium Carbonate‐Binding Protein, Blue Mussel Shell Protein (BMSP), from the Nacreous Layer. *Chembiochem* **12**, 2478-2487 (2011).

4 Mann, K., Edsinger-Gonzales, E. & Mann, M. In-depth proteomic analysis of a mollusc shell: acid-soluble and acid-insoluble matrix of the limpet Lottia gigantea. *Proteome science* **10**, 28 (2012).

5 Marie, B. *et al.* The shell‐forming proteome of Lottia gigantea reveals both deep conservations and lineage‐specific novelties. *The FEBS journal* **280**, 214-232 (2013).

6 Takeuchi, T. *et al.* Bivalve-specific gene expansion in the pearl oyster genome: implications of adaptation to a sessile lifestyle. *Zoological letters* **2**, 3 (2016).

7 Simakov, O. *et al.* Insights into bilaterian evolution from three spiralian genomes. *Nature* **493**, 526 (2013).

8 Luo, Y.-J. *et al.* The Lingula genome provides insights into brachiopod evolution and the origin of phosphate biomineralization. *Nature communications* **6**, 8301 (2015).

9 Song, X., Wang, X., Li, L. & Zhang, G. Identification two novel nacrein-like proteins involved in the shell formation of the Pacific oyster Crassostrea gigas. *Molecular biology reports* **41**, 4273-4278 (2014).

10 Kazusa, D. N. A. R. I. *et al.* Sequence and analysis of chromosome 5 of the plant Arabidopsis thaliana. *Nature* **408**, 823-826, doi:10.1038/35048507 (2000).

11 Montgomery, J. C., Venta, P. J., Tashian, R. E. & Hewett-Emmett, D. Nucleotide sequence of human liver carbonic anhydrase II cDNA. *Nucleic acids research* **15**, 4687-4687, doi:10.1093/nar/15.11.4687 (1987).

12 Nagao, Y., Platero, J. S., Waheed, A. & Sly, W. S. Human mitochondrial carbonic anhydrase: cDNA cloning, expression, subcellular localization, and mapping to chromosome 16. *Proceedings of the National Academy of Sciences* **90**, 7623-7627 (1993).

13 Fujikawa-Adachi, K., Nishimori, I., Taguchi, T. & Onishi, S. Human Mitochondrial Carbonic Anhydrase VB cDNA CLONING, mRNA EXPRESSION, SUBCELLULAR LOCALIZATION, AND MAPPING TO CHROMOSOME X. *Journal of Biological Chemistry* **274**, 21228-21233 (1999).

14 Aldred, P. *et al.* Human secreted carbonic anhydrase: cDNA cloning, nucleotide sequence, and hybridization histochemistry. *Biochemistry* **30**, 569-575 (1991).

15 [Pastorek, J.](https://www.uniprot.org/uniprot/?query=author:%22Pastorek+J.%22&sort=score), [Pastorekova, S.](https://www.uniprot.org/uniprot/?query=author:%22Pastorekova+S.%22&sort=score), [Callebaut, I.](https://www.uniprot.org/uniprot/?query=author:%22Callebaut+I.%22&sort=score), [Mornon, J. P.](https://www.uniprot.org/uniprot/?query=author:%22Mornon+J.-P.%22&sort=score), [Zelnik, V.](https://www.uniprot.org/uniprot/?query=author:%22Zelnik+V.%22&sort=score), [Opavsky, R.](https://www.uniprot.org/uniprot/?query=author:%22Opavsky+R.%22&sort=score), [Zat'Ovicova, M.](https://www.uniprot.org/uniprot/?query=author:%22Zat%27Ovicova+M.%22&sort=score), [Liao, S.](https://www.uniprot.org/uniprot/?query=author:%22Liao+S.%22&sort=score), [Portetelle, D.](https://www.uniprot.org/uniprot/?query=author:%22Portetelle+D.%22&sort=score), [Stanbridge, E. J.](https://www.uniprot.org/uniprot/?query=author:%22Stanbridge+E.J.%22&sort=score), [Zavada, J.](https://www.uniprot.org/uniprot/?query=author:%22Zavada+J.%22&sort=score), [Burny, A.](https://www.uniprot.org/uniprot/?query=author:%22Burny+A.%22&sort=score) & [Kettmann, R.](https://www.uniprot.org/uniprot/?query=author:%22Kettmann+R.%22&sort=score) **Cloning and characterization of MN, a human tumor-associated protein with a domain homologous to carbonic anhydrase and a putative helix-loop-helix DNA binding segment.** *Oncogene* **9**, 2877-2888 (1994).

16 Ota, T. *et al.* Complete sequencing and characterization of 21,243 full-length human cDNAs. *Nature genetics* **36**, 40 (2004).

17 Albertin, C. B., Simakov, O., Mitros, T., Wang, Z. Y., Pungot, J. R., Edsinger-Gonzalez, E., Brenner, S., Ragsdale, C. W., Rokhsar, D. S. WGS assembly of Octopus bimaculoides. EMBL/GenBank/DDBJ databases (2015).

18 Song, X. & [Wang, X.](https://www.uniprot.org/uniprot/?query=author:%22Wang+X.%22&sort=score) **Three novel carbonic anhydrases from the mantle of the Pacific oyster (Crassostrea gigas) are the duplication after the oyster species formation.** EMBL/GenBank/DDBJ databases (2013).
